# High-resolution ex vivo NMR spectroscopy of human Z α_1_-antitrypsin

**DOI:** 10.1038/s41467-020-20147-7

**Published:** 2020-12-11

**Authors:** Alistair M. Jagger, Christopher A. Waudby, James A. Irving, John Christodoulou, David A. Lomas

**Affiliations:** 1 UCL Respiratory, Rayne Institute, University College London, London, WC1E 6JF UK; 2 Institute of Structural and Molecular Biology, University College London and School of Crystallography, Birkbeck College, University of London, Gower Street, London, WC1E 6BT UK

**Keywords:** Protein aggregation, Glycoproteins, Solution-state NMR, Molecular medicine

## Abstract

Genetic mutations predispose the serine protease inhibitor α_1_-antitrypsin to misfolding and polymerisation within hepatocytes, causing liver disease and chronic obstructive pulmonary disease. This misfolding occurs via a transiently populated intermediate state, but our structural understanding of this process is limited by the instability of recombinant α_1_-antitrypsin variants in solution. Here we apply NMR spectroscopy to patient-derived samples of α_1_-antitrypsin at natural isotopic abundance to investigate the consequences of disease-causing mutations, and observe widespread chemical shift perturbations for methyl groups in Z AAT (E342K). By comparison with perturbations induced by binding of a small-molecule inhibitor of misfolding we conclude that they arise from rapid exchange between the native conformation and a well-populated intermediate state. The observation that this intermediate is stabilised by inhibitor binding suggests a paradoxical approach to the targeted treatment of protein misfolding disorders, wherein the stabilisation of disease-associated states provides selectivity while inhibiting further transitions along misfolding pathways.

## Abstract (web summary)

α_1_-Antitrypsin (AAT) is a 52 kDa serum glycoprotein, the misfolding and polymerisation of which is associated with COPD and liver disease. Here the authors demonstrate the use of high-resolution multidimensional solution-state NMR spectroscopy to characterise the structure and dynamics in solution of Z AAT purified directly from clinical patients.

## Introduction

**Fig. 1:**
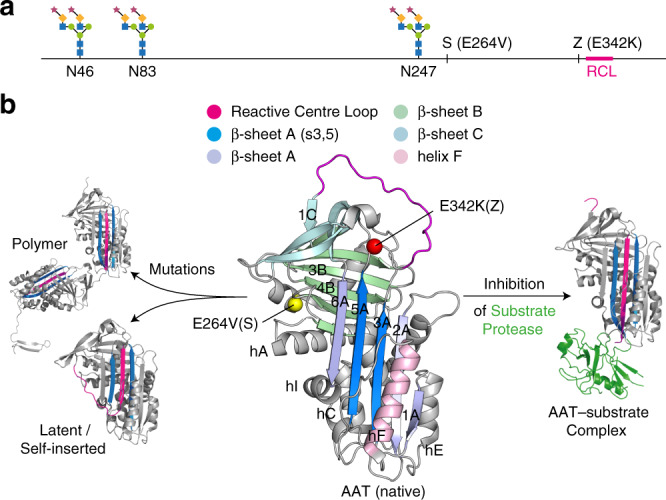
Structure, function and polymerisation of AAT. **a** Schematic indicating the position of *N*-linked glycans (with the abundant M6 glycoform shown)^45^, disease-associated mutations and the reactive centre loop. **b** Crystal structures of recombinant AAT highlighting key structural features and the location of disease-associated mutations, and the large-scale conformational changes associated with function and dysfunction: 1QLP^72^, 1EZX^73^, 1IZ2^74^ and an adaptation of 3T1P^7^.

α_1_-Antitrypsin (AAT) is a 394 residue (52 kDa) glycoprotein (Fig. 1a) present at high concentrations (1–2 g L^−1^) in human plasma, where it primarily functions as an inhibitor of neutrophil elastase^1,2^. As with other members of the serpin family of protease inhibitors, AAT has a metastable fold and an exposed reactive centre loop (RCL) that inserts into the central β-sheet A upon cleavage by the target protease to form an inactive protease–inhibitor complex (Fig. 1b)^3^. However, spontaneous self-insertion of the RCL can lead to formation of an inactive latent form or ordered polymer chains by insertion into other AAT molecules (Fig. 1b)^4–7^.

Point mutations of AAT can promote misfolding and polymerisation within the hepatocyte secretory apparatus, leading to cellular stress, intrahepatic accumulation of intractable protein deposits and predisposition to liver cirrhosis^8–10^. This accumulation causes a concomitant lack of circulating plasma AAT that leads to unregulated neutrophil elastase activity within the lung and early onset chronic obstructive pulmonary disease^11^.

Of the variants responsible for severe AAT deficiency, the Z allele (E342K), present in 1–4% of descendants of Northern European Caucasian populations, accounts for over 95% of clinical cases, and in homozygotes results in a ca. 85–90% decrease in circulating AAT levels. The mild deficiency S allele (E264V) is carried by up to 15% of southern Europeans, and in homozygotes results in a decrease of ca. 40%^12,13^. The names of common AAT alleles follow an historic nomenclature, in which they are assigned letters based on their migration during isoelectric focussing electrophoresis with respect to the wild-type M (medium) allele^12^. In this work, this single-letter nomenclature will refer exclusively to ex vivo human material, whereas recombinant samples will be explicitly labelled as such.

The ability of the Z mutation to render AAT prone to misfolding and polymerisation has been studied using a variety of biophysical techniques. The opening of strands 3 and 5 in β-sheet A sheet is a necessary precursor to either functional or pathological loop insertion (Fig. 1b), and the loss of a salt bridge between E342 at the base of the RCL and K290 in strand 6 of β-sheet A in the Z variant has been proposed to destabilise the molecule and facilitate structural rearrangements in the upper breach region of β-sheet A, close to the site of the mutation^3^. The existence of such an activated intermediate state, in dynamic equilibrium with the native state and populated more readily in Z than in wild-type AAT, has been indicated by several lines of evidence including far-UV circular dichroism, fluorescence spectroscopy and extrinsic dye binding^14^, accessibility of engineered cysteine residues to covalent modification^15,16^, and an increased incorporation rate of a RCL peptide mimetic in Z AAT^17^. However, the structure of this intermediate remains controversial, being variously described as comprising one or multiple states, and as folded^18^, near-native^19^, molten globule^20^, or partially unfolded^7^. Moreover, a crystal structure of Z AAT expressed by *Drosophila* S2 cells is essentially indistinguishable from the wild-type structure (all-atom rmsd 0.6 Å, Supplementary Fig. 1) and as such fails to elucidate the basis for its aberrant behaviour^16^. We speculated that this structure may not fully reflect the range of conformations sampled by Z AAT in solution, and therefore turned to solution-state NMR spectroscopy in order to investigate further the structure and dynamics of AAT variants.

NMR spectroscopy provides an array of methods to study protein structure, dynamics and interactions, across a range of timescales, in both the polypeptide backbone and in sidechains^21–23^. We have previously reported NMR observations of recombinant AAT, including the near-complete assignment of the polypeptide backbone using ^1^H,^15^N-TROSY methods in perdeuterated AAT^24–26^. However, our and others’ attempts to recombinantly express the E342K (Z) AAT variant in a system compatible with isotopic enrichment have failed^27^, owing to a high propensity to misfold and aggregate during expression, and reduced stability in the absence of glycosylation^28^. Therefore, in this work we have investigated the acquisition of high-resolution two-dimensional (2D) NMR spectra of sidechain methyl groups using patient-derived samples of AAT variants at natural isotopic abundance, in order to probe the solution structure and dynamics of AAT variants in their natively glycosylated forms.

## Results

### Ex vivo NMR spectroscopy of AAT variants

Biomolecular NMR spectroscopy is typically performed today using recombinantly expressed protein samples prepared using a variety of isotopic (^2^H, ^13^C, ^15^N) enrichment schemes^29–31^. Such labelling serves multiple purposes: first, ^15^N or ^13^C labelling permits the resolution of many otherwise overlapping resonances from amide or methyl groups using multidimensional spectroscopy; and second, substitution of ^2^H for ^1^H eliminates undesirable relaxation pathways, facilitating the study of larger, more slowly tumbling molecules. Even in the absence of enrichment however, ^15^N and ^13^C nuclei are present at low levels (0.37% and 1.1%, respectively), permitting heteronuclear NMR experiments to be acquired at natural isotopic abundance^32–36^. Such experiments present a number of technical challenges, described further below, but importantly the natural abundance NMR approach provides a route to study systems such as disease-associated AAT variants that are resistant to recombinant expression and isotopic labelling. Therefore, a series of samples of ex vivo M, Z and S AAT were prepared from the plasma of healthy individuals (M AAT) or from patient donors genotyped to be homozygous for Z or S alleles. Owing to low levels of circulating AAT in patients homozygous for the Z allele, plasma was pooled from 13 individuals prior to purification in order to obtain sufficient material for further investigation.

**Fig. 2:**
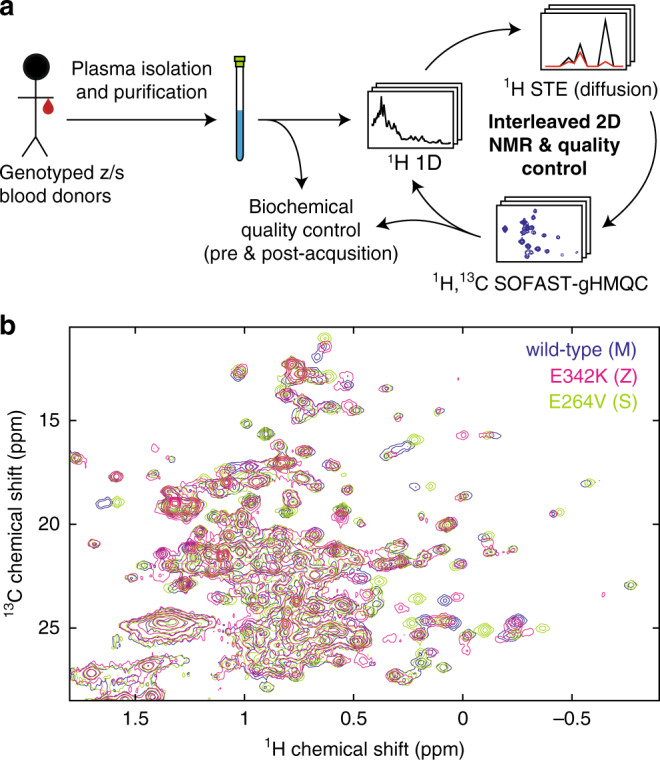
Ex vivo NMR spectroscopy of AAT. **a** Overview of ex vivo AAT sample preparation, data acquisition and quality control strategy for ex vivo NMR. **b** ^1^H,^13^C SOFAST-gHMQC spectra (Supplementary Fig. 5) acquired for ex vivo wild-type (M), Z and S AAT variants (298 K, 900 MHz). Contour levels are normalised for concentration and acquisition time.

Biomolecular NMR at natural abundance poses challenges in terms of sensitivity and sample stability, spectral resolution and background suppression. Optimisation of sensitivity is a trade-off between the concentration and temperature of the sample (higher temperatures accelerate rotational diffusion leading to reduced relaxation and hence increased sensitivity), and its stability over long acquisition times, particularly for aggregation-prone systems such as AAT. Following a series of exploratory measurements, we selected a sample concentration of 400 μM (~10–25-fold higher than that of wild-type levels of circulating AAT in human plasma^13^) at 298 K, under which conditions we found that all variants remained stable over an acquisition period of ca. 80 hr. No changes were observed between 1D ^1^H spectra acquired at AAT concentrations of 1 and 400 µM, indicating that the elevated concentrations used in this study did not induce detectable changes such as oligomerisation (Supplementary Fig. 2). Following protocols developed for the analysis of unstable ribosome–nascent chain complexes^37^, ^1^H 1D and translational diffusion measurements^38^ were acquired every 5 hr during the NMR acquisition period to provide a real-time assessment of sample stability (Fig. 2a). In addition, sample integrity was assayed pre- and post-acquisition by native and denaturing PAGE and functional assays of inhibitory activity. All samples remained >90% monomeric and functional throughout the measurements (Supplementary Fig. 3).

Although we have previously used ^15^N-based experiments to study AAT^24–26^, in the present work we employed ^13^C-based experiments owing to its greater natural abundance and properties exhibited by methyl groups that make them favourable for use in challenging systems. In particular, we focused on the study of ILVMA sidechain methyl groups, which are widespread in AAT and so provide excellent coverage across the entire structure. Rapid rotation about the methyl threefold symmetry axis leads to the methyl-TROSY effect, which strongly suppresses certain relaxation pathways within the ^13^CH_3_ spin system of the methyl group, thus facilitating the study of large, slowly tumbling molecules^39^. As this effect is independent of the magnetic field strength, to maximise resolution we utilised the highest available field strength for our measurements (900 MHz ^1^H Larmor frequency). However, although the methyl-TROSY effect is optimised within the HMQC (heteronuclear multiple quantum correlation) experiment, multiple quantum coherences present during the ^13^C chemical shift encoding period are susceptible to relaxation by dipolar interactions with other protons, and this is particularly significant when observing large, fully protonated molecules^39^. In contrast, the HSQC (heteronuclear single quantum correlation) experiment is less optimal in terms of the methyl-TROSY effect, but single quantum coherences present during the ^13^C chemical shift encoding period are more resistant to relaxation by external spins^39^. We therefore carried out preliminary measurements to compare optimised HMQC and HSQC experiments and found, at least in our particular case, that the HMQC provided comparable resolution and greater sensitivity than the equivalent HSQC experiment (Supplementary Fig. 4).

To obtain further increases in experimental sensitivity, we employed the longitudinal relaxation-optimised methyl-SOFAST-HMQC experiment^40^. This experiment employs selective ^1^H pulses to avoid perturbing non-observed spins, and so is particularly effective for fully protonated samples such as ex vivo AAT, which provide an abundant bath of spins to accelerate the recovery of magnetisation between scans. However, when applied to natural abundance measurements we found that large levels of *t*_1_ noise were present, owing to imperfect cancellation of signals across the phase cycle, which hindered further analysis. We therefore developed a gradient-selected variant of the experiment, the methyl-SOFAST-gHMQC (Supplementary Fig. 5). Although the introduction of gradient selection resulted in a √2 reduction in the sensitivity of the experiment, *t*_1_ noise was effectively eliminated, leading to higher quality spectra. Therefore, this experiment was employed for all further measurements.

Having established optimised sample conditions and experimental parameters, a series of 2D ^1^H,^13^C methyl-SOFAST-gHMQC correlation spectra were acquired for M, Z and S AAT variants, providing high-resolution fingerprints of methyl groups distributed throughout AAT (Fig. 2b). Even in the absence of detailed resonance assignments, the similarity between these spectra indicated that all variants were fully folded and have broadly similar structures in solution. However, to enable a more detailed, residue-specific analysis of these spectra, we next undertook an assignment of AAT methyl resonances.

### Assignment of AAT methyl resonances

The limited sensitivity of NMR at natural isotopic abundance precludes the assignment of ex vivo samples by direct approaches. We therefore followed a two-step strategy, in which methyl resonances were first assigned using samples of isotopically labelled recombinant AAT, and then these assignments were transferred to resonances in ex vivo spectra.

**Fig. 3:**
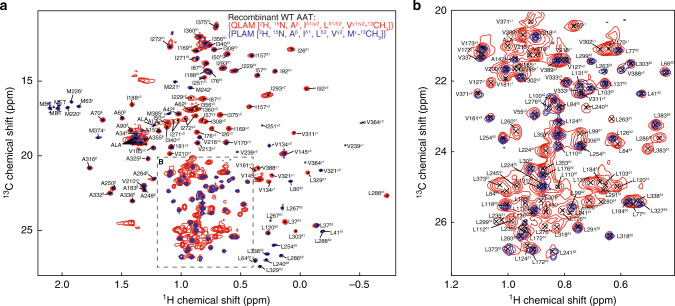
Assignment of AAT methyl resonances. **a** Assigned ^1^H,^13^C correlation spectra of recombinant wild-type AAT, prepared with two complementary methyl-labelling schemes as indicated. **b** Magnified region of **a**.

To maximise coverage of methyl groups, perdeuterated samples of recombinant wild-type AAT were prepared using two isotopic labelling schemes. Firstly, a sample (QLAM) was prepared in which A^β^, I^γ2/δ1^, L^δ1^ and V^γ1^ methyl groups were ^13^CH_3_ labelled and connected to the polypeptide backbone along linearised ^13^C chains, and L^δ2^ and V^γ2^ methyl groups were ^13^CH_3_ labelled and connected to ^13^CD_3_ L^δ2^ and V^γ2^ methyl groups along a linear ^13^C chain^41^. Using a series of 3D COSY experiments, this enabled the identification of residue types, the unambiguous and stereospecific pairing of multiple methyls within single I, L and V residues, and the transfer of assignments from the existing backbone assignment of AAT^24^ by correlation of CO, Cα and Cβ chemical shifts^42,43^. Second, a sample (PLAM) was prepared with ^13^CH_3_ labelling of A^β^, I^δ1^, L^δ2^, V^γ2^ and M^ε^ methyl groups, for assignment of methionine resonances and the verification of ambiguous QLAM assignments using through-space magnetisation transfer experiments (4D SOFAST-HMQC-NOESY-SOFAST-HMQC). In total, this resulted in a final assignment coverage of 97.1% (19 of 19 I^δ1^, 19 of 19 I^γ2^, 45 of 45 L^δ1^, 45 of 45 L^δ2^, 23 of 24 V^γ1^, 24 of 24 V^γ2^, 16 of 22 A^β^, 7 of 9 M^ε^) (Fig. 3).

Resonance assignments were then transferred from recombinant (wild-type) AAT to spectra of ex vivo AAT variants (Supplementary Fig. 6–8) using an iterative, structure-based strategy. Resonances that remained unperturbed between recombinant AAT and ex vivo AAT spectra were assigned first, followed by the transfer of assigned peaks in the recombinant AAT spectrum to resonances within 0.05 ppm (^1^H) or 0.1 ppm (^13^C). Assignments were then transferred for resonances exhibiting larger chemical shift perturbations (CSPs) based on the following principles: the nearest neighbours in coordinate space, as defined in a reference crystal structure (1QLP), to sites exhibiting perturbed resonances are also most likely to be perturbed; the largest CSPs are likely to occur closest to sites of modification by glycosylation or mutation; conversely, residues distant from these positions are not expected to be perturbed; and roughly similar degrees of perturbation are expected for both resonances in residues with bearing more than one methyl group (i.e., Ile, Leu and Val). This process was iterated until self-consistent solutions were reached (Supplementary Fig. 6–8).

### Comparison of ex vivo M and recombinant wild-type AAT

**Fig. 4:**
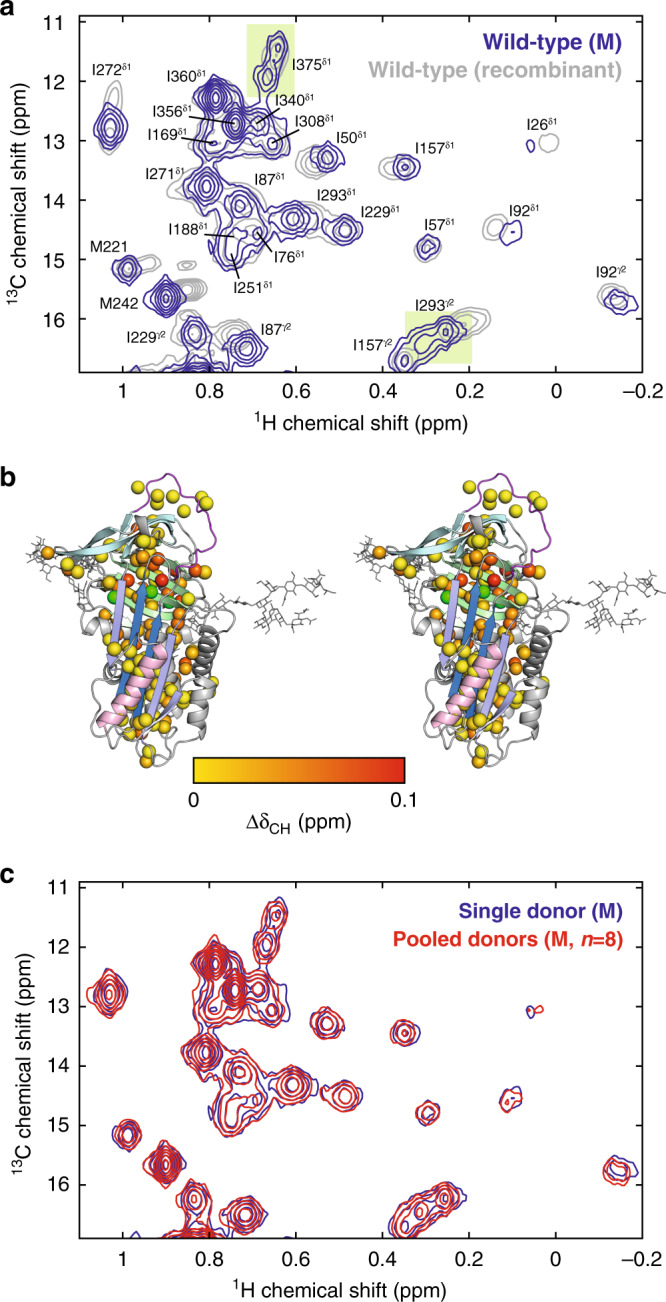
Comparison of ex vivo M and recombinant WT AAT. **a** Comparison of ^1^H,^13^C SOFAST-gHMQC spectra (isoleucine regions) of M AAT and U-^1^H,^13^C recombinant wild-type AAT (deconvolved using NUWS-NUS^44^) (298 K, 900 MHz). Resonance assignments are indicated, and green shading highlights the presence of doubled peaks. **b** Stereo view of combined methyl chemical shift changes, ${\Delta}\delta_{{\mathrm{CH}}} = \sqrt {{\Delta}{\delta}_{\mathrm{H}}^2 + \left( {\Delta}\delta_{\mathrm{C}}/4 \right)^2}$, projected onto the AAT structure^72^ with the most abundant M6 isoform glycans shown^45^, modelled using CHARMM-GUI^75^. Doubled methyl resonances are indicated in green. **c** Comparison of ^1^H,^13^C SOFAST-gHMQC spectra (isoleucine region) of ex vivo M AAT purified from a single donor and from a pool of eight donors.

Having assigned methyl resonances, we first sought to compare the spectrum of natively glycosylated ex vivo M AAT with the (non-glycosylated) recombinant wild-type (Fig. 4a, Supplementary Fig. 6). As the spectra used for resonance assignment were obtained from perdeuterated protein with residue-specific methyl-labelling, to enable a comparison of both peak positions and resonance intensities with the spectrum of ex vivo M AAT, a sample of fully protonated, uniformly ^13^C-labelled recombinant AAT was expressed and a reference spectrum acquired using an identical methyl-SOFAST-gHMQC pulse sequence and a NUS-NUWS homonuclear decoupling scheme^44^.

The spectrum of M AAT purified from a single donor overlapped extensively with that of non-glycosylated recombinant AAT (Fig. 4a and Supplementary Fig. 6). As methyl chemical shifts are sensitive reporters of the local chemical environment, this observation by itself indicates these proteins have near-identical solution structures. Nevertheless, small CSPs (Δδ_CH_ < 0.04 ppm) were observed in methyl groups approximately adjacent to the N47, N83 and N247 glycosylation sites (Fig. 4b and Supplementary Fig. 9), indicative of small perturbations to the local chemical environment at these positions relative to non-glycosylated recombinant AAT.

*N*-linked glycosylation of AAT within the ER results in a variety of glycoforms, which can be resolved in isoelectric focusing (IEF) gels (Supplementary Fig. 3e)^45^. Given the observation above of CSPs associated with glycosylation, we investigated the extent to which different glycoforms might give rise to distinct CSPs. We identified just three such doubled peaks, which we attribute to I293^γ2^, M374, and I375^δ1^ resonances, all of which are in the proximity of the N247 glycosylation site (Fig. 4a, b). In contrast, only single resonances were observed in recombinant AAT. To investigate this further, we compared our spectrum of M AAT purified from a single donor with that of M AAT purified from the pooled plasma of eight donors (Fig. 4c and S10). Although differences in isoelectric focussing banding patterns confirmed that the glycosylation profile of these samples differed (Supplementary Fig. 3e), the spectra from single and pooled sources were almost indistinguishable, with no discernible changes in peak number, chemical shifts or intensities. We therefore conclude that glycan heterogeneity has a very limited impact on the core protein structure, and that the variation that does exist arises within an individual rather than between multiple donors. Importantly, this observation validates our later use of pooled samples for disease-associated AAT variants, for which sufficient material could not be obtained from single patient donors.

Next, we examined the variation in resonance intensities between methyl groups in the recombinant wild-type and ex vivo M AAT spectra. Such site-to-site variations reflect differences in transverse relaxation rates, which in turn are determined primarily by the local proton density, the rate of rotational diffusion (represented by the rotational correlation time, τ_c_), and the local mobility at a particular site (represented by an *S*^2^ order parameter), although chemical exchange (i.e., exchange between multiple conformations having distinct chemical shifts) on an approximately micro–millisecond timescale may also contribute to increased relaxation rates and hence reduced resonance intensities. Therefore, the analysis of resonance intensities provides a valuable tool for probing conformational dynamics and the potential existence of intermediate states. However, in the case of M AAT, while resonance intensities were variable they were inversely correlated with the effective rotational times, *S*^2^τ_c_, measured in a sample of recombinant AAT (Supplementary Fig. 9c). This indicates that they are determined primarily by rotational diffusion of the molecule and local mobility, with no evidence of additional relaxation due to chemical exchange with a second conformational state.

### Comparison of M and Z AAT

**Fig. 5:**
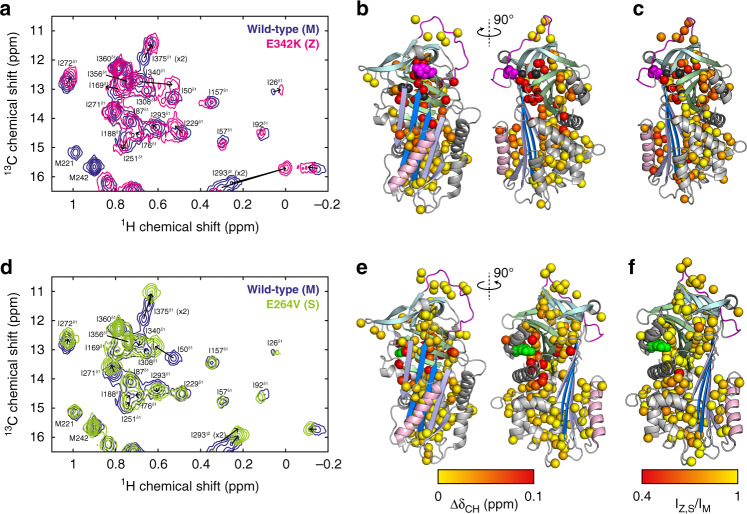
Comparison of wild-type (M), Z and S AAT variants. **a**–**c** Comparisons of ex vivo NMR observations of M AAT with those of the Z variant, and **d**–**f** with the S variant. **a**, **d** Overlays of ^1^H,^13^C SOFAST-gHMQC spectra (isoleucine region) for M, Z and S AAT, with resonance attributions indicated. Contour levels are normalised for concentration and acquisition time. **b**, **e** Combined methyl chemical shift changes between wild-type and variant AAT, ${\Delta}\delta_{{\mathrm{CH}}} = \sqrt {{\Delta}{\delta}_{\mathrm{H}}^2 + \left( {\Delta}\delta_{\mathrm{C}}/4 \right)^2}$, projected onto the wild-type structure^72^. Resonances whose attribution could not be traced are indicated in dark grey, and mutations in the Z and S variants are indicated with magenta and green spheres, respectively. Colouring of secondary structure elements is consistent with Fig. 1b. **c**, **f** Intensity changes between M and Z/S variants of AAT, projected onto the wild-type recombinant AAT structure^72^.

Having investigated the impact of glycosylation on M AAT relative to the recombinant wild-type, we next sought to characterise the structural and dynamic perturbations to AAT induced in solution by the disease-associated Z variant, by comparing its ^1^H,^13^C correlation spectra with that of the M variant (Fig. 5a and Supplementary Fig. 7). However, in addition to the E342K mutation, the Z allele also carries the V213A mutation, found in rare M isoforms and not associated with misfolding or disease^46^. To determine whether this mutation induces CSPs not attributable to the Z mutation, we expressed recombinant V213A AAT and recorded its ^1^H,^13^C correlation spectrum, but only limited CSPs were observed relative to the recombinant wild-type, which were localised to the site of the mutation (Supplementary Fig. 11). Consequently, we attribute the additional perturbations described below to the primary E342K mutation alone.

A comparison of ex vivo M and Z AAT spectra revealed widespread chemical shift perturbations (Fig. 5b). In contrast to M AAT, no doubled peaks were observed for I293^γ2^, M374, I375^δ1^ or any other resonance in Z AAT, indicating that the impact of glycan heterogeneity on the core protein structure is even more limited than observed for M AAT. The largest CSPs between M and Z AAT were observed for methyl groups in close proximity to the E342K mutation, at the top of strand 5 in β-sheet A (Fig. 5b and Supplementary Fig. 12). Local perturbations such as these are to be expected and report on direct changes to the local chemical environment arising from the mutation. In contrast, resonances of residues within the RCL (Fig. 1) were not perturbed, which indicates that the RCL of Z AAT does not partially insert into sheet A, as previously speculated^47^.

Equally, however, we also observed moderately large chemical shift perturbations (${\mathrm{{\Delta}}}\delta _{{\mathrm{CH}}} > 0.1$ ppm), extending across a wide region of the AAT beyond the immediate vicinity of the Z mutation, and particularly towards the helix F/post-helix F loop and the underlying region of strands 3 and 5 of β-sheet A (Fig. 5b). This region is a central component of the serpin inhibitory apparatus, as large conformational changes must occur in both the F helix and β-sheet A to enable insertion of the RCL within the sheet, and it has also been implicated by multiple studies in the polymerisation mechanism^6–8,17,48^ (Fig. 1b). Although it is not possible to identify the detailed structural changes associated with these perturbations, the observed CSPs nevertheless indicate that there is a long-range propagation of structural perturbations beyond the site of mutation. We suggest that these perturbations are likely to reflect alternative sidechain packing arrangements, which may facilitate further, larger conformational rearrangements on a slower timescale.

Lastly, we have compared resonance intensities between M and Z AAT. After accounting for minor differences in sample concentration and acquisition time, we found that many resonances had a significantly lower intensity in Z AAT (Fig. 5c and Supplementary Fig. 12), indicating that these resonances must have increased relaxation rates. Although it is possible that this reflects reduced mobility (i.e., higher effective rotational correlation times) in the Z variant, we have also observed that such resonances are also associated with larger CSPs. This suggested instead that increased relaxation rates may be owing to chemical exchange between multiple conformations, thus providing an initial indication, explored further below, that the observed CSPs may be associated with dynamic processes rather than simply static perturbations to the AAT structure.

### Comparison of M and S AAT

In comparison with Z AAT, the ^1^H,^13^C correlation spectrum of S AAT matched that of M AAT much more closely, with CSPs observed only in regions close to the site of the mutation (Fig. 5d, e and Supplementary Fig. 13). It was previously suggested that the S mutation may lead to altered sidechain packing around β-sheet A, in particular at the key strands 3 and 5, which form a shutter to accept a donor strand during substrate inhibition or polymerisation^49^. However, the absence of CSPs in this region—which would be sensitive to altered sidechain packing and rotamer distributions—disfavours this possibility. Peak intensities also remained unchanged relative to M AAT, indicating that the S mutation also does not alter the conformational dynamics of the molecule, at least on the ca. millisecond timescale accessible through these measurements (Fig. 5f and Supplementary Fig. 13).

### Characterisation of the interaction of AAT variants with a small-molecule inhibitor of polymerisation

**Fig. 6:**
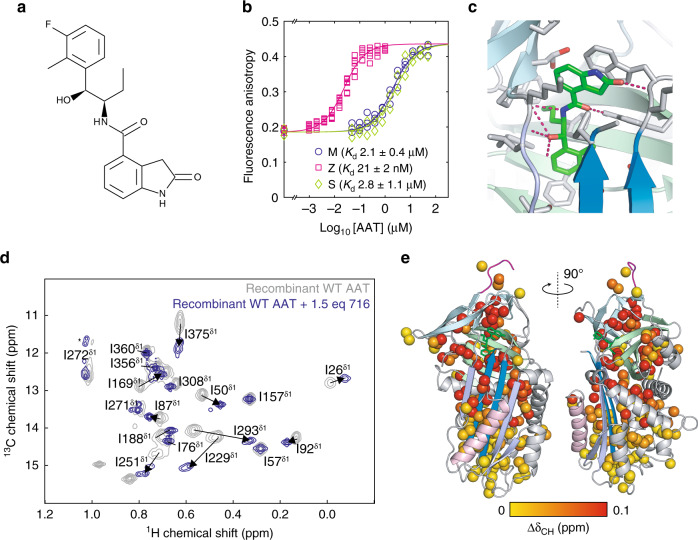
Binding of the small-molecule polymerisation inhibitor 716 to AAT. **a** Chemical structure of the inhibitor 716^50^. **b** Measurement of the binding affinity of Alexa-488-labelled 716 for M, Z and S AAT using a fluorescence polarisation assay^50^. **c** Crystal structure (PDB ID: 7AEL) illustrating the binding site of 716^50^. **d** Assigned ^1^H,^13^C HMQC spectra (isoleucine region) of [^2^H, A^β^I^δ1^L^δ2^V^γ2^M^ε^-^13^CH_3_]-labelled recombinant wild-type AAT in the presence and absence of an excess of 716. Asterisks indicate minor peaks arising from sample degradation. **e** Combined methyl chemical shift changes in recombinant AAT upon 716 binding, ${\Delta}\delta_{{\mathrm{CH}}} = \sqrt {{\Delta}{\delta}_{\mathrm{H}}^2 + \left( {\Delta}\delta_{\mathrm{C}}/4 \right)^2}$, projected on the structure of 716-bound AAT^50^. The bound 716 ligand is indicated in green. Colouring of secondary structure elements is consistent with Fig. 1b. Source data are provided as a Source Data file.

We next used ex vivo NMR to investigate the interaction of AAT variants with a small molecule, GSK716 (716), that is a potent inhibitor of AAT polymerisation in vitro, in cells, and in a mouse model of AAT deficiency (Fig. 6a)^50^. This molecule binds close to the site of the Z mutation, E342K, with a significantly higher affinity for Z AAT than for M or S AAT (Fig. 6b–c)^50^.

We first investigated the interaction of 716 with recombinant wild-type AAT, and observed large and widespread CSPs (Fig. 6d). As binding occurred in slow exchange on the NMR chemical shift timescale, consistent with a slowly dissociating, high-affinity ligand, resonance assignments could not be tracked from free to bound states. We therefore carried out a de novo assignment of bound resonances using 4D NOESY measurements of two samples, comprising first uniform ^13^CH_3_ labelling of ILV methyl groups, and second with stereospecific ^13^CH_3_ labelling of A^β^, I^δ1^, L^δ2^, V^γ2^ and M^ε^ methyl groups (Fig. 6d and Supplementary Fig. 14). As expected, we found that the largest CSPs occurred in methyl groups in direct proximity to the ligand binding site, but significant CSPs (${\mathrm{{\Delta}}}\delta _{{\mathrm{CH}}} > 0.1$ ppm) were still observed as distantly as the F helix, >25 Å away (Fig. 6e and Supplementary Fig. 15).

**Fig. 7:**
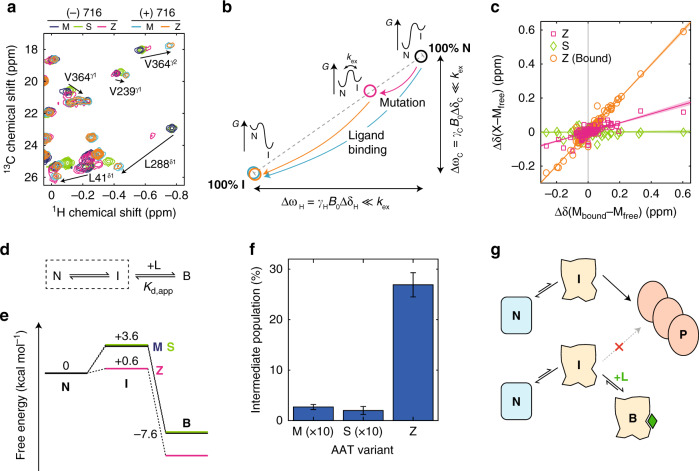
Quantification of intermediate populations in ex vivo AAT variants. **a** Overlays of ^1^H,^13^C SOFAST-gHMQC spectra (isoleucine region) for free M (blue), Z (magenta) and S (green) AAT, and 716-bound M (cyan) and Z (orange) AAT, with selected resonance attributions and colinear chemical shift perturbations indicated. Contour levels are normalised for concentration and acquisition time. **b** Schematic illustration of the origin of collinear chemical shift perturbations, arising from a rapid equilibrium between native (N) and intermediate (I) states. **c** Chemical shift perturbations of AAT resonances relative to wild-type M AAT, plotted against chemical shift changes in M AAT upon 716 binding. Lines of best fit through the origin are shown. ^1^H and ^13^C chemical shift differences are plotted independently and ^13^C measurements have been scaled by a factor of 0.25. Resonances near sites of mutation or 716 binding showing evidence of local perturbations to their chemical environment have been excluded from this analysis. **d** The conformation selection reaction scheme. A dotted box highlights the intramolecular equilibrium, while the apparent dissociation constant of the ligand is determined relative to the total concentration of unbound species, $K_{\mathrm{{d,app}}}=([\mathrm{N}]+[\mathrm{I}])/[\mathrm{B}]$. **e** Free-energy diagram of AAT showing native (N), intermediate (I) and 716-bound (B) states under standard conditions. **f** Populations of the intermediate state in wild-type and variant AAT. Error bars indicate the standard error determined from fits. **g** Cartoon illustration of the inhibition of AAT misfolding and polymerisation (P) via the binding and stabilisation of an on-pathway intermediate state by a small-molecule ligand. Source data are provided as a Source Data file.

^1^H,^13^C correlation experiments were then acquired for M and Z AAT in the presence of saturating concentrations of 716 (Fig. 7a, Supplementary Fig. 16, 17). In contrast to spectra of M and Z AAT acquired in the absence of 716, these spectra were extremely similar, indicating that ligand binding stabilises both variants in a common structural state. Doubled peaks observed in the M *apo* state were also found to merge into single resonances in the bound state, implying that even these minor structural variations that we have attributed to glycan heterogeneity are eliminated upon 716 binding.

### Correlated chemical shift perturbations identify a transient intermediate in Z AAT

Resonance assignments were transferred from the 716-bound recombinant spectrum to bound ex vivo M and Z AAT spectra using the iterative, structure-based approach described earlier. Subsequent spectral overlays revealed that the CSPs observed between unbound M and Z AAT were collinear with those observed upon binding of 716, indicating that changes in the methyl ^1^H and ^13^C chemical shifts were strongly correlated. Moreover, this correlation was observed across many methyl groups, with Z AAT resonances observed at an approximately constant fraction of the distance between the unbound M and bound M/Z resonances (Fig. 7a).

Collinear, correlated CSPs are a powerful and unambiguous indicator of conformational exchange and arise where a rapid equilibrium exists between native (N) and intermediate (I) states with distinct chemical shifts, such that the observed chemical shift is the population-weighted average of the individual states (i.e., fast exchange on the chemical shift timescale). Modulation of this equilibrium, either by mutation or by ligand binding, thus results in correlated perturbations across many resonances, which in 2D spectra gives rise to collinear shift perturbations (Fig. 7b)^51^. Analysis of such CSPs can therefore be used to determine changes in the populations of native and intermediate states relative to a reference point, which we choose here to be wild-type (M) AAT. Using linear regression, the extent of CSPs between M and Z or S AAT was found to be 27 ± 2% (Z, ± s.e.) and 0.4 ± 1.7% (S, ± s.e.) of those observed in wild-type (M) AAT upon 716 binding (Fig. 7c). In this analysis, methyl groups directly adjacent either to the site of a mutation or to the ligand binding site were excluded, as these exhibited large additional perturbations arising from direct changes to their local environment.

In the case of AAT, as 716 binding is observed to shift the equilibrium towards the intermediate state (Fig. 7a) we conclude that 716 does not bind to the native state but must instead interact through a conformational selection mechanism (Fig. 7d), in which an intermediate conformation that is spontaneously sampled by AAT is stabilised by ligand binding. In this model, the ligand has a constant affinity for the intermediate state across all variants, and so the apparent macroscopic affinity is determined only by the equilibrium population of the intermediate in the absence of the ligand. As we observe the population of this intermediate to be significantly increased by the Z mutation, this accounts for the observed increase in the macroscopic affinity of 716 for the Z variant (Fig. 6b).

To quantify the intermediate populations in AAT variants, we fitted our observations of relative CSPs, together with measurements of 716 binding affinities (Fig. 6b), to a conformational selection scheme (Fig. 7d) in order to determine both the affinity of 716 for the pure intermediate state, and the relative chemical shift of the pure native state (see Methods). Given the high affinity of the ligand for AAT, together with the fact that binding resulted in very similar chemical shifts for both M and Z AAT, we have assumed that the bound state shifts are a close approximation to those of the (unbound) intermediate. The populations of the intermediate state at equilibrium could then be determined from the observed CSPs, which also allowed the relative free energies of native (N), intermediate (I) and 716-bound (B) states in M, Z and S AAT to be calculated (Fig. 7e–f).

This description is consistent with the observation that relative affinities for 716 are determined primarily by different rates of association, reflecting in turn the different intermediate state populations in each variant^50^. The increased intermediate population we find for Z AAT also accounts for the observation that this variant incorporates a peptide mimetic of the RCL into β-sheet A more rapidly than M AAT^17^. Perturbations in Z AAT encompass key regions of the protein, including the F helix and strands 3 and 5 of β-sheet A, that are implicated in polymerisation and that are associated with other pathogenic mutations^52^. However, although the CSPs are widespread, as they are limited in magnitude we further conclude that the intermediate has a near-native conformation, in contrast to the extensively perturbed conformations proposed elsewhere^6,7^.

The progressive changes in chemical shift observed between resonances in M, Z and ligand-bound AAT (Fig. 7a) also indicate that the rate of exchange between native and intermediate states, *k*_ex_, must be much greater than the frequency difference between the states, Δ*ω* = *γB*_0_Δδ, where *B*_0_ is the magnetic field strength and *γ* is the gyromagnetic ratio of the observed nucleus^53^. As the largest observed frequency difference is Δ*ω* = 2250 s^−1^ for L288^δ1^_H_, we therefore infer that the intermediate is sampled rapidly, on a timescale much shorter than 500 μs (*τ*_ex_ ≪ 1/Δ*ω*).

## Discussion

Two-dimensional biomolecular NMR at natural isotopic abundance was originally developed several decades ago for the analysis of highly concentrated solutions of relatively low molecular weight proteins, prior to the development and widespread adoption of isotopic labelling techniques^32,33^. A profusion of advanced labelling methods have now been developed, primarily using recombinant expression in bacterial cells^29,30^, but also using yeast, insect and mammalian cells^54–56^. Nevertheless, challenging systems remain that are not amenable to recombinant expression and labelling, or that contain posttranslational modifications that are hard to reproduce. Meanwhile, improvements in the sensitivity and field strength of NMR spectrometers, together with advances in pulse sequence design, have opened up new possibilities for the analysis of large, unlabelled biomolecules at natural isotopic abundance, and this has been applied, for example, to monitor higher order structure of therapeutic proteins and biosimilars^35^.

In this work, we have shown that NMR at natural abundance can also be applied effectively to the analysis of protein samples purified from patients, possessing the full range of posttranslational modifications and therefore truly representative of the material involved in disease. From this, we have been able to identify the transient species associated with a serpin-mediated conformational disorder. Such ex vivo approaches have developed extensively in cryo-EM in recent years^57^, but solution-state NMR spectroscopy brings an unparalleled ability to characterise dynamic or disordered systems at high resolution.

Using ex vivo NMR, we have observed CSPs associated with an altered ensemble of conformations in Z AAT with respect to M AAT. As there is close correspondence between the X-ray crystal structures of these variants^16^, the conformation of Z AAT in solution must therefore be distinct from that observed in the crystal lattice. Instead, we have shown that the observed perturbations are associated with the increased sampling of an intermediate state, accessed on a sub-millisecond timescale, and, unexpectedly, that this intermediate is directly stabilised by the binding of the small-molecule inhibitor of polymerisation, 716.

These findings have a number of implications. First, our observations indicate that the structure of the AAT intermediate must resemble that of 716-bound AAT. A crystal structure of this state (Fig. 6c) revealed that 716 binds to a hydrophobic pocket, which displays altered sidechain packing and distortion at the top of strand 5 A (Fig. 1b)^50^. Our data demonstrate that this interaction is the product of conformational selection rather than an induced fit. Accordingly, in the structure of the ligand-unbound intermediate, this surface-accessible pocket would be expected to be present but occupied by solvent. This is consistent with the observation that one of its constituent residues, Trp194, is more solvent exposed in Z AAT^58^ and that an E342A substitution at the site of the Z mutation induces lability in the top of β-strand 5A^16^. Disorder in this breach region is likely to result in aberrant interactions between the RCL and β-sheet A, resulting in misfolding or polymerisation. We note also that the intermediate is not populated significantly more in S AAT than in M AAT, which correlates with the lack of accumulation of polymers in the liver of S/S homozygotes^59^.

Following cleavage of the RCL by a target protease, inhibition is a race between unproductive hydrolysis of the acyl-enzyme intermediate, and RCL insertion and protease inactivation, which occurs on a timescale of ca. 1 s^60^. It is therefore interesting to note that Z AAT displays both an increased intermediate population and reduced inhibitory activity (as determined by the stoichiometry of inhibition), as distortion in the breach region could be expected to facilitate the opening of β-strands 5A and 3A and thereby permit rapid incorporation of the RCL. However, a nascent β-hairpin-like backbone conformation present at the top of β-strand 5A in the native structure is lost in the intermediate (as represented by the 716-bound structure). As this β-hairpin becomes the secondary structural element adjoining β-strand 5A and the inserting β-strand 4A during the inhibition of the protease, its loss would likely result in a lack of coordination at this point of initial strand insertion. This incompatibility of the intermediate conformation with effective inhibition is consistent with the apparent correspondence between its 27% population by Z AAT, determined here, and the ca. 70% inhibitory activity of the variant with respect to M AAT^61^—reflecting a similar 30% of non-productive interactions with a target protease. From these observations, it may be hypothesised that the lesser inhibitory activity of the Z variant is driven by a binary exchange between an inhibition-competent native conformation and an inactive intermediate conformation that does not lie on the functional pathway of substrate inhibition.

Nevertheless, the observation that the intermediate is directly stabilised by binding of the small-molecule inhibitor of polymerisation 716 provides a direct connection between our experiments and a molecular change that underpins AAT deficiency in situ: as the compound is an active inhibitor of polymerisation both in vivo and in vitro^50^, the intermediate conformation we identify must also be present and directly involved in pathological processes in the liver. This observation also suggests a paradoxical approach to the targeted treatment of protein misfolding disorders: by stabilisation of the pathological intermediate state, rather than the native state, selectivity for disease-associated variants may be achieved while nevertheless thermodynamically and kinetically inhibiting transitions out of this state along the disease pathway.

## Methods

### Preparation of ex vivo AAT

Human ex vivo AAT variants were collected from the blood of consenting donors in the presence of 10% (w/v) sodium citrate anticoagulant. Plasma was isolated by centrifugation at 1200 × *g* for 30 mins at 4 °C. The supernatant containing blood plasma was removed and subjected to a second round of centrifugation. Samples were stored at −80 °C until required. Frozen plasma was defrosted on ice and centrifuged twice at 3000 × *g* for 30 mins at 4 °C to remove aggregates formed upon thawing. Cleared plasma was filtered through a glass wool fibre pre-filter and then again through a 0.45 μm filter before loading on to a column containing Alpha-1-Antitrypsin-select resin (Cytiva) equilibrated in 20 mM Tris, 150 mM NaCl, pH 7.4. The column was washed with ca. 10 volumes of equilibration buffer then AAT was eluted with the addition of 2 M MgCl_2_. Fractions containing AAT were dialysed into 20 mM Tris, pH 8.0, 0.02% (w/v) NaN_3_, reduced with 100 mM 2-mercaptoethanol (BME) for 10 mins then loaded onto an equilibrated HiTrap Q HP ion-exchange column (GE Healthcare) and eluted over a gradient from 0 to 500 mM NaCl. Fractions containing AAT were dialysed into 25 mM sodium phosphate, 50 mM NaCl, 0.02% (w/v) NaN_3_, pH 7.6, purified by size exclusion chromatography, then stored at −80 °C prior to use^62^. For Z AAT samples, plasma of 13 patients was pooled prior to the first purification step.

Samples were from patients attending a specialised Alpha-1-Antitrypsin deficiency clinic at the Royal Free Hospital, London. Ethics oversight: REC reference 13/LO/1085—NHS Health Research Authority NRES Committee London-Hampstead.

### Preparation of recombinant AAT

Recombinant AAT protein constructs were cloned into the PQE30 vector and include an N-terminal hexa-histidine tag for purification. BL21 cells (NewEngland BioLabs, Ipswich MA, USA) were co-transformed with both the AAT PQE30 plasmid and a pREP4 plasmid containing the *lac* repressor. The QuikChange site-directed mutagenesis kit (Qiagen) was used to introduce single point mutations as per manufacturer’s instructions with *Escherichia coli* plasmid propagation in XL1-Blue (Agilent) supercompetent cells. Non-isotopically labelled AAT was expressed and then purified using ion-exchange and size exclusion chromatography as described above.

Uniformly ^1^H,^13^C-labelled AAT was prepared by growing *E. coli* in an M9-based expression medium (EM9)^63^. BL21 cells were grown to stationary phase in LB medium (Sigma Aldrich). Cells were collected by centrifugation at 4000 × *g* for 20 min and resuspended in EM9 medium at OD_600_ 0.01, containing ^12^C-glucose (0.4% (w/v)). After growth (18–20 h, 37 °C, 250 rpm), cells were harvested by centrifugation and transferred to EM9 medium containing ^13^C_6_-d-glucose (0.2% (w/v)) at OD_600_ 0.05 and grown to OD_600_ ~ 0.6, induced with 1 mM IPTG and expressed for 16 h at 24 °C, 250 rpm.

[^2^H, ILV-^13^CH_3_]-labelled AAT was expressed using BL21 cells progressively adapted into EM9 media prepared with 99.8% D_2_O and ^12^C-d_7_-D-glucose (0.2% w/v). BME vitamins were substituted with Yeast Nitrogen Base without amino acids (BD Difco) at 0.8 g/L. Cells were initially grown to stationary phase in LB medium (Sigma Aldrich) then collected by centrifugation (4000 × *g*, 20 min) and resuspended in 5 mL EM9 medium (80% D_2_O) at OD_600_ 0.01. After growth (18–20 h, 37 °C, 250 rpm), cells were again harvested by centrifugation and transferred to 5 mL EM9 medium (~99.8% D_2_O) at OD_600_ 0.01. After growth (8 h, 37 °C, 250 rpm), cells were harvested and transferred to 5 mL EM9 medium (~99.8% D_2_O, 0.2% (w/v) ^12^C-d_7_-glucose) at OD_600_ 0.01 and grown for 18–20 h, 37 °C, 250 rpm. Finally, cells were harvested and used to inoculate a large volume of EM9 medium (99.9% D_2_O, 0.2% (w/v) d_7_-glucose) at OD_600_ 0.05. The culture was grown at 37 °C with shaking until OD_600_ ~ 0.6, at which point 80 mg/L of 2-ketobutyric acid sodium salt (methyl-^13^C, 99%; 3,3-d_2_ 98%) and 160 mg/L of 2-ketoisovaleric acid sodium salt (3-methyl-^13^C, 99%; 3,4,4,4-d_4_) were added. The culture was incubated for 1 h at 30 °C and then induced with 1 mM isopropyl β- d-1-thiogalactopyranoside (IPTG) (prepared in D_2_O) and expressed for 16 h at 24 °C. Cells were harvested by centrifugation and AAT purified as described above. Isotopes were purchased from Cambridge Isotopes (Tewksbury, MA, USA).

[^2^H, ^15^N, A^β^I^δ1^L^δ2^M^ε^V^γ2^-^13^CH_3_]-labelled AAT and [^2^H, ^15^N, A^β^I^δ1/γ2^L^δ1/2^V^γ1/2^-^13^CH_3_]-labelled AAT were expressed using PLAM and QLAM isotopic labelling kits (NMRBio, Grenoble, France) according to the manufacturer’s protocol. Cultures were grown at 37 °C with shaking until OD_600_ ~ 0.8, then induced with 1 mM IPTG (prepared in D_2_O) for 16 h at 24 °C and harvested and purified as described above.

### Sodium dodecyl sulphate polyacrylamide gel electrophoresis

The NuPAGE Bis-Tris gel system (Life Technologies, Warrington, UK) was used following the manufacturer’s instructions. Typically samples containing 4 μg protein were mixed with NuPAGE lithium dodecyl sulphate (LDS) sample buffer (1× final concentration) containing either 1,4-dithiothreitol (DTT) (10 mM final concentration) or BME (50 mM final concentration) and heated at 90 °C for 5 min. Gels were visualised by staining with Instant blue Coomassie stain (Expedeon, Cambridge, UK).

### Non-denaturing (native) polyacrylamide gel electrophoresis

Native gels were used to evaluate the monomeric and oligomeric state of AAT. The Novex Bis-Tris 3–12% (w/v) acrylamide gel system (Life Technologies, Warrington, UK) was used following the manufacturer’s instructions. Protein samples were mixed 1:1 with sample buffer (50% (v/v) glycerol containing bromophenol blue) containing either DTT (10 mM final concentration) or BME (50 mM final concentration) and loaded at 4 μg protein per lane. Gels were visualised by staining with Instant blue Coomassie stain (Expedeon, Cambridge, UK).

### Isoelectric focusing

Novex pre-Cast isoelectric focusing (IEF) gels (pH 3–7) (Life Technologies, Warrington, UK) were used to determine the isoforms of the ex vivo AAT material following the manufacturer’s instructions. Samples containing 4 μg protein were mixed with 5 μL Novex IEF sample buffer and diluted to 10 μL using dH_2_O. Samples were loaded on to the gel alongside the IEF migration markers and run using Novex IEF cathode buffer and IEF anode buffer at 100 V constant for 1 h, followed by 200 V constant for 1 h, followed by 500 V constant for 30 mins, at 4 °C. The gel was fixed using 12% (v/v) trichloroacetic acid solution for 30 mins at room temperature before staining with Instant blue Coomassie stain (Expedeon, Cambridge, UK).

### Protease activity assays

AAT activity was quantified through the stoichiometry of inhibition (SI), which gives a measure of the number of molecules of AAT required to inhibit one molecule of protease^64^. Protease inhibition is a 1:1 reaction, therefore an SI value of 1 indicates fully active AAT for M and S variants. Z AAT is a less-efficient protease inhibitor, with a reported SI value of 1.5:1^65^. Aliquots of between 0 and 10 μL AAT (2 µM) in protease assay buffer (20 mM Tris, 100 mM NaCl, 0.1% (w/v) PEG 8000, 10 mM CaCl_2_ pH 8.0) were diluted to a volume of 10 μL; 10 µL of 50 nM bovine α-chymotrypsin in protease assay buffer was then added and incubated for 15 mins at 25 °C. From a 222 µM stock, 180 μL of chromogenic α-chymotrypsin substrate *N*-succinyl-Ala-Ala-Pro-Phe-p-nitroanilide substrate (Calbiochem) was then added and the change in absorbance at 405 nm was recorded over 5 mins using a SpectraMax M5e plate reader (Molecular Devices) to determine residual α-chymotrypsin activity. Linear regression was used to extrapolate the amount of inhibitor required to completely abrogate enzyme activity. The inhibitory activity of ex vivo AAT samples was determined pre- and post-NMR acquisition.

### Measurement of 716 affinity

An Alexa-Fluor488-labelled variant of the 716 ligand (AF488-716) was used to obtain an estimate of the affinity of unlabelled 716 for M, S and Z AAT using fluorescence polarisation (FP). M and S AAT were titrated from 100 and 50 μM, respectively, in a one in two dilution series over 12 points in PBS with 0.01% (v/v) Tween 20, in a half-area, black, μClear microplate (Greiner) with a final assay volume of 40 μL. Z AAT was titrated from 1 μM. AF488-716 (10 nM final concentration) was added for 30 mins at room temperature and FP determined using a Molecular Devices Spectramax M5e plate reader, with excitation and emission wavelengths of 490 and 525 nm, respectively, with excitation cutoff filter at 515 nm at 25 °C. Titration data were fitted in MATLAB to determine the dissociation constants of AF488-716 for AAT variants (*n* = 2–4 independent measurements).

### NMR spectroscopy

NMR data were acquired at 298 K on Bruker Avance III or Avance III HD spectrometers equipped with TXI or TCI cryogenic probes operating at field strengths from 600 to 950 MHz (^1^H Larmor frequency) and using TopSpin 3.6. Data were processed and analysed using nmrPipe^66^ version 9.9, CCPN Analysis^67^, NMR-FAM Sparky^68^ and GraphPad Prism version 6.0 f for Mac.

NMR samples of recombinant, isotopically labelled AAT were prepared in 25 mM Na_2_HPO_4_, pH* 7.6, 50 mM NaCl, 1 mM EDTA, 1 mM DTT, 0.02% (w/v) NaN_3_, 100% D_2_O. Samples of ex vivo AAT variants and recombinant ^1^H, ^13^C WT AAT were prepared in 25 mM Na_2_HPO_4_, pH 8.0, 50 mM NaCl, 1 mM EDTA, 1 mM DTT, 0.02% (w/v) NaN_3_, 10% D_2_O (v/v).

For recombinant samples, ^1^H,^13^C-HMQC correlation spectra were typically acquired using 256 points with a sweep width of 17.5 ppm in the indirect ^13^C dimension, and 4096 points and a sweep width of 20 ppm in the direct, ^1^H dimension. Spectra were recorded with a 1 s inter-scan delay and were processed using cosine-squared window functions.

Ex vivo samples (422 μM) were acquired at 900 MHz (21.1 T). ^1^H,^13^C-SOFAST-gHMQC experiments (Supplementary Fig. 5) were acquired using 4096 points with a sweep with of 22 ppm in the direct dimension, and 320 points and a sweep width of 40 ppm in the indirect dimension, corresponding to acquisition times of 103 ms and 18 ms, respectively. Spectra were recorded with a 300 ms inter-scan delay and were processed with linear prediction using cosine-squared window functions. For comparison, spectra of fully protonated, uniformly ^13^C-labelled AAT (104 μM) were acquired with identical acquisition parameters, using a non-uniform weighted sampling scheme and processed as non-uniform sampled data in order to eliminate the 35 Hz homonuclear ^1^*J*_CC_ coupling^44^. For experiments collected in the presence of 716 ligand dissolved to 50 mM in d_6_-DMSO (98%), a twofold molar excess of ligand to protein was added to AAT variants and incubated for a minimum of 30 min at 25 °C then acquired as described for samples in the absence of ligand. A vehicle control was performed by 1D NMR, adding an equivalent volume of d_6_-DMSO (98%) (~1.7%) to ex vivo M AAT and comparing the spectrum to an equivalent sample in the absence of DMSO (Supplementary Fig. 18).

### Diffusion measurements and analysis

The oligomeric state of ex vivo AAT variants was assessed throughout the duration of NMR experiments by interleaved ^1^H STE diffusion experiments^38^. These were recorded using an encoding/decoding gradient length, *δ*, of 4 ms, a diffusion delay, Δ, of 100 ms, and 24 points at gradient strengths between 2% and 98% of the maximum, 55.57 G cm^−1^. The signal in the methyl region was integrated at each gradient strength and the diffusion coefficient calculated using the Stejskal-Tanner equation by non-linear fitting in dosyView (nmrPipe). Error bars report the standard error of the diffusion coefficient determined from the non-linear fit.

### *S*^*2*^τ_c_ measurements

The mobility on a ps–ns timescale of individual methyl groups was assessed by measurements of the product *S*^*2*^τ_c_, where τ_c_ is the effective rotational correlation time of AAT, and *S*^*2*^ is an order parameter for the orientation of the methyl symmetry axis. Low values of *S*^*2*^τ_c_ therefore indicate more mobile regions of the molecule. S^2^τ_c_ values were determined by measurement of the rate of cross-correlated relaxation-induced build-up of triple quantum coherences^69^. Triple quantum build-up and single quantum relaxation measurements were acquired at 950 MHz with delays of 1.1, 3, 6, 10, 15, 25, and 40 ms, and peak intensities were then determined using nmrPipe and fitted to determine *S*^*2*^τ_c_ values^69^.

### Assignment of methyl resonances

Stereospecific assignment of ILVMA methyl resonances in native and 716-bound AAT was carried out using selective labelling, through-bond magnetisation transfer experiments, through-space magnetisation transfer (NOESY) experiments and mutagenesis, in combination with computational structure-based assignment software (MAGMA)^70^.

Three-dimensional HMCM(CG), HMCMCG(CB), and HMCMCGCB(CA) experiments were used to assign QLAM {Ala^β^, Ile^δ1/γ2^, Leu^δ1/2^, Val^γ1/2^} U-[^15^N,^13^C,^2^H] labelled samples by chemical shift correlation with the published backbone assignment (BMRB ID:17804)^24^. Experiments were recorded using a 900 MHz spectrometer following a divide-and-conquer strategy where the chemical shift of individual carbons are collected in separate experiments rather than simultaneously^42,43^. For carbonyl carbon assignment experiments, HMCM(CA)CO, HMCM(CBCA)CO, and HMCM(CGCBCA)CO experiments were acquired at 600 MHz to reduce the impact of chemical shift anisotropy on the transverse relaxation of carbonyl carbons. Spectra were acquired with 4096 points and sweep width of 20 ppm in the direct, ^1^H dimension; 128 points and sweep width of 19 ppm in the ^13^C_methyl_ dimension; and 64 points and sweep width of 14 ppm in the ^13^CO dimension; corresponding to acquisition times of ~102, ~13, and ~9 ms, respectively. A 2 s inter-scan delay was used and spectra were processed using cosine-squared window functions.

Four-dimensional SOFAST-HMQC-NOESY-SOFAST-HMQC experiments were used to assign ^2^H-Ile^δ1^, Leu^δ1/2^, Val^γ1/2^-[^13^CH_3_] (ILV), and PLAM {Ala^β^, Ile^δ1^, Leu^δ2^, Met^ε^, Val^γ2^} U-[^15^N,^12^C,^2^H] labelled samples in apo and holo forms. For the holo assignment, 716 ligand was added in d_6_-DMSO (98%) to recombinant labelled samples at a twofold molar excess and incubated for 30 min at 25 °C prior to acquisition. Experiments were acquired at 900 MHz, with 4096 points and a sweep width of 20 ppm in the direct dimension; 128 points and a sweep width of 18 ppm in the ^13^C_methyl_ dimension; 128 points and a sweep width of 18 ppm in the ^13^C_NOE_ dimension; and 128 points and a sweep width of 2.4 ppm in the ^1^H_NOE_ dimension, corresponding to acquisition times of ~114, ~16, ~16, and ~25 ms, respectively. Experiments were acquired using NUS with 20,000 hyper-complex points (with a sparsity of 7.6%), a 400 ms mixing time and 200 ms recycle delay, and were reconstructed using SMILE2.0b^71^. Spectra were viewed in MATLAB (R2015b, The MathWorks Inc.) (https://github.com/chriswaudby/4d-viewer). Some preliminary resonance assignments were obtained using MAGMA^70^ (with addition of code to account for stereospecific labelling). These were subsequently verified and further assignments determined by manual inspection of NOE data and conservative mutagenesis.

### Assignment of ex vivo AAT spectra

Assignments were transferred to resolved resonances in ex vivo NMR spectra of AAT variants by a spectral overlay with the assigned recombinant protein in both free and bound forms, using an iterative, structure-based attribution process assuming (i) that the largest chemical shift perturbations are expected closest to sites of glycosylation, mutation or ligand binding, (ii) conversely, residues distant from these sites are not expected to be perturbed, and (iii) that the nearest neighbours to perturbed sites (based on the X-ray crystal structure of wild-type AAT) are also likely to be perturbed. Assignment transfers were validated through correlation of observed chemical shift changes with regions of perturbation between apo/holo X-ray crystal structures^50,72^ and through correlation of resonance intensities and linewidths. Resonances that remained unperturbed between recombinant AAT and ex vivo AAT spectra were assigned first. Transfers were then made to unassigned resonances that were within ≤0.05 ppm (^1^H dimension) or ≤0.1 ppm (^13^C dimension) of an assigned peak in the spectra of recombinant AAT. Where assignments were transferred to resonances with CSPs greater than those mentioned above, these were verified to be residues close to site of mutation/ligand binding. In addition, in these cases, a similar degree of chemical shift perturbation was expected for the methyl resonance of each spin system for residues with more than one methyl group. For the spectrum of Z AAT, the new location of five residues (M221, M242, A250, and L291) could not be determined (Fig. 5b). From X-ray crystal structure analysis, all residues had sidechains that protrude into the pocket at the site of the Z mutation.

### Determination of intermediate state populations

Linear regression was used to determine the extent of chemical shift perturbations in Z and S AAT, relative to the chemical shift perturbations observed in wild-type (M) AAT upon 716 binding. ^1^H and ^13^C chemical shift perturbations within a methyl group were analysed independently, with ^13^C measurements being scaled by a factor of 0.25 to reflect the difference in gyromagnetic ratios of the two nuclei. Methyl groups directly adjacent either to the site of a mutation or to the ligand binding site were excluded from analysis, as these exhibited large additional perturbations arising from direct changes to the local chemical and magnetic environment.

Data were fitted to a conformational selection scheme in which ligand binding occurs via the intermediate state: ${\mathrm{N}}\mathop { \rightleftharpoons }\limits^K {\mathrm{I}} \mathop { \rightleftharpoons }\limits^{K_{\mathrm{d}}\;( + {\mathrm{L}})} {\mathrm{B}}$ (Fig. 7d, e). The equilibrium constant for formation of the intermediate in variant X, $K_{\mathrm{X}}$, can be expressed in terms of the observed chemical shift, $\delta _{\mathrm{X}}$, the chemical shift of the intermediate, which is equivalent to that of the bound state, $\delta _{\mathrm{B}}$ (after excluding resonances exhibiting large local perturbations), and the (unknown) chemical shift of the native state, $\delta _{\mathrm{N}}$:

1 $$\begin{array}{*{20}{c}} {K_{\mathrm{X}} = \frac{{p_{\mathrm{I}}^{\mathrm{X}}}}{{p_{\mathrm{N}}^{\mathrm{X}}}} = \frac{{\delta _{\mathrm{X}} - \delta _{\mathrm{N}}}}{{\delta _{\mathrm{B}} - \delta _{\mathrm{X}}}}} \end{array}$$

The experimentally observed dissociation constant for binding of ligand to an AAT variant can also be expressed in terms of this equilibrium constant, and the dissociation constant, $K_{\mathrm{d}}$, for direct binding to the intermediate state:

2 $$\begin{array}{*{20}{c}} {K_{{\mathrm{d,}}\,{\mathrm{obs}}}^{\mathrm{X}} = \frac{{1 + K_{\mathrm{X}}}}{{K_{\mathrm{X}}}}K_{\mathrm{d}}}\end{array}$$

These expressions were fitted globally for all AAT variants to determine $\delta _{\mathrm{N}}$ and $K_{\mathrm{d}}$ values, from which the populations of the intermediate state could be determined (Fig. 7f), as well as a complete thermodynamic description of the system (Fig. 7e).

### Reporting summary

Further information on research design is available in the Nature Research Reporting Summary linked to this article.

## Supplementary information

Supplementary Information

Peer Review File

Reporting Summary

## Data Availability

The data that support this work are available from the corresponding authors upon reasonable request. Chemical shift assignments and time-domain NMR data for ex vivo samples have been deposited in the Biological Magnetic Resonance Data Bank (https://bmrb.io/) with accession codes 50530 to 50536. Protein structures used in this study can be found in the PDB with the accession codes 1QLP (native AAT)^72^ and 7AEL (716-bound AAT)^50^. Source data are provided with this paper.

## Source data

Source data
