# Supplementary material for: High-resolution ex vivo NMR spectroscopy of human Z α1-antitrypsin
Source: Nat Commun. 2020 Dec 11;11:6371. doi: 10.1038/s41467-020-20147-7 (PMC7732992; doi:10.1038/s41467-020-20147-7)
Supplement: Supplementary file 1 — Supplementary Information [file 41467_2020_20147_MOESM1_ESM.pdf]

High-resolution *ex vivo* NMR spectroscopy of human Z α_1-antitrypsin

Alistair M. Jagger*, Christopher A. Waudby*, James A. Irving, John Christodoulou and David A. Lomas

Supplementary Figures

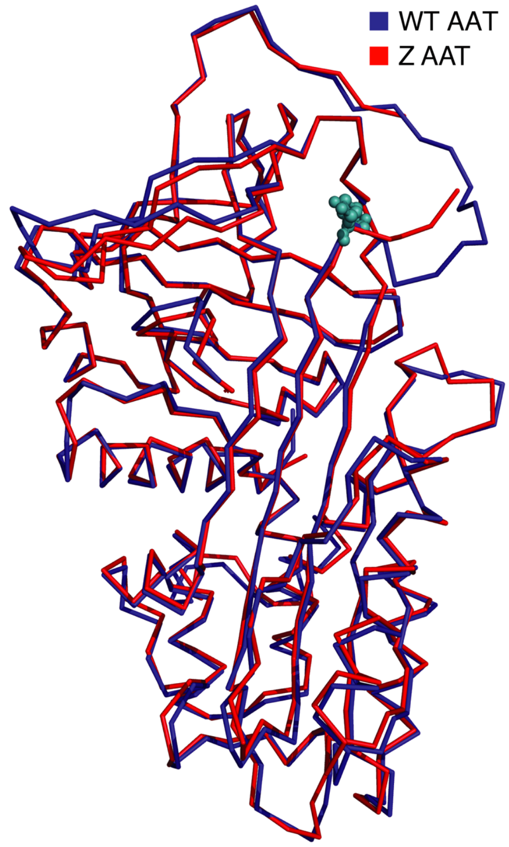

Fig. Supplementary 1: Comparison of X-ray crystal structures of WT and Z AAT. Overlay of X-ray crystal structures of WT AAT (PDB ref 1QLP) (blue) and Z AAT (PDB ref 5iO1) (red) shown as line representation, with the position of the Z mutation shown as colored atoms (teal). (All-atom rmsd 0.6 Å).

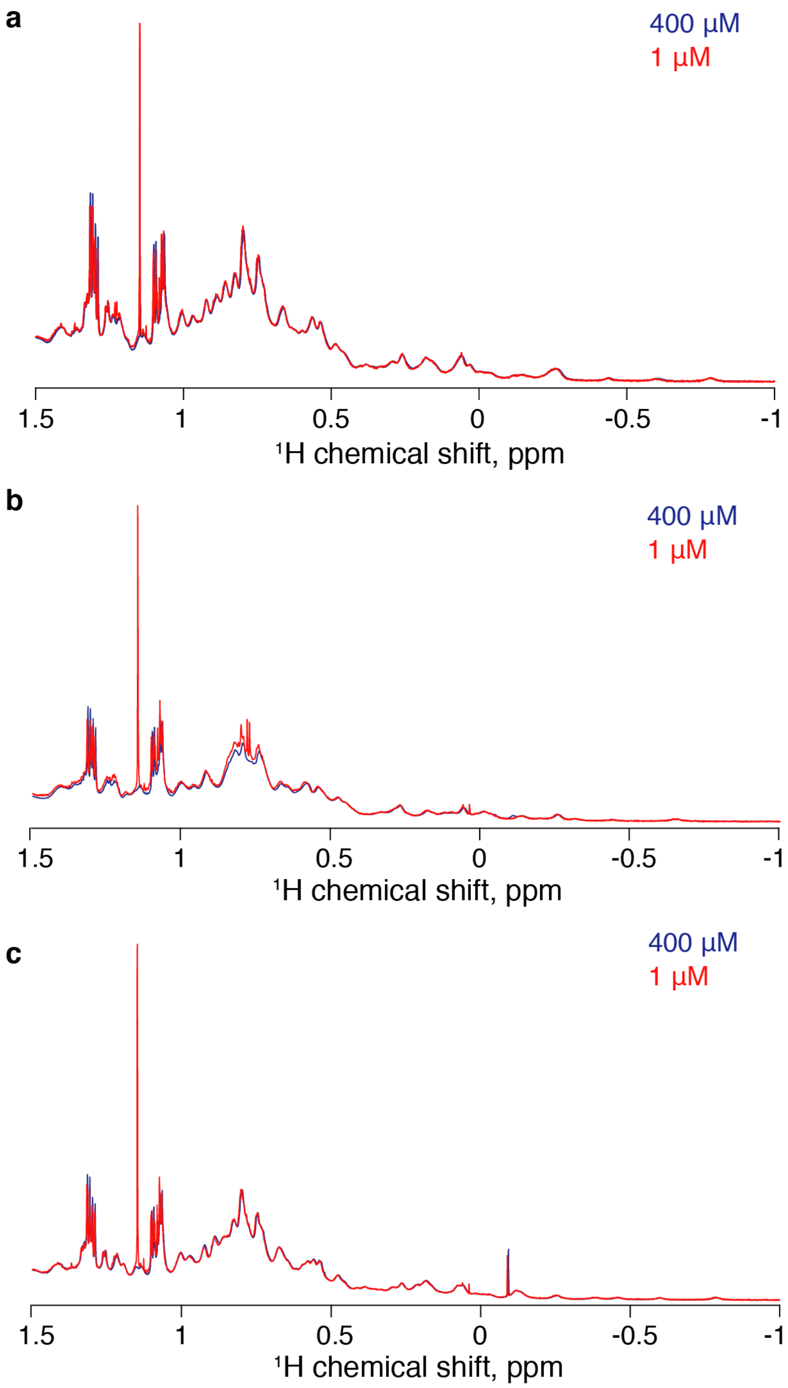

Fig. Supplementary 2: Concentration dependence of ^{1}H 1D spectra of *ex vivo* AAT variants. Methyl resonances are shown, normalized for concentration and acquisition time, for (a) M, (b) Z and (c) S AAT, at the concentrations indicated.

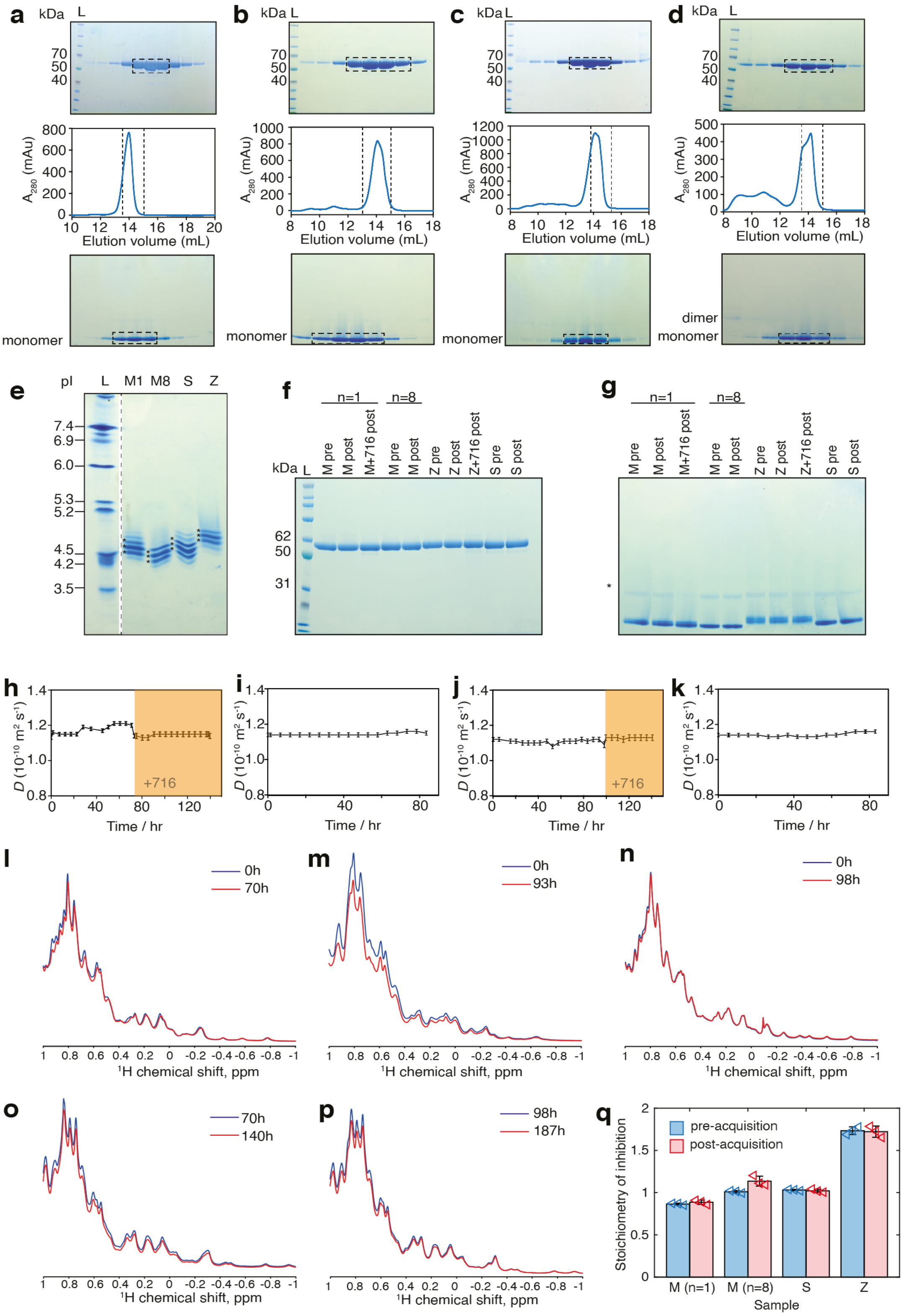

Fig. Supplementary 3: Preparation and quality control of *ex vivo* AAT variants for NMR. *Ex vivo* AAT variants were prepared from human plasma using a three-step purification as described in materials and methods. Gel-filtration purification traces are shown (middle panel) for **(a)** M AAT from one individual, **(b)** M AAT pooled from the plasma of 8 individuals, **(c)** Z AAT pooled from the plasma of 13 individuals, and **(d)** S AAT from the plasma of one individual. Vertical dotted lines

indicate the fractions used for the NMR sample. Upper and lower panels show SDS PAGE and native PAGE gels respectively to show purity and oligomeric state of samples used for NMR. L indicates molecular weight ladder. The position of monomeric and dimer components are indicated on native PAGE gels. Dashed boxes correspond to the region between dashed lines in the chromatography trace. **(e)** Isoelectric focusing (IEF) over a pH range 3-7 for M1 (individual donor), M8 (8 pooled donors), S (individual donor) and Z (13 pooled donors) AAT variants shows altered charge distribution of each variant. Asterisks mark the major glycoforms in each sample. L indicates IEF ladder (pH 3-10) with approximate isoelectric point (pI) positions shown. **(f)** SDS PAGE of NMR samples collected pre- and post-NMR acquisition and in presence of 716 ligand as indicated. L indicates molecular weight ladder with the approximate masses of bands shown. **(g)** Native PAGE of NMR samples collected pre- and post-NMR acquisition and in the presence of 716 ligand as indicated. Asterisk indicates a small amount of oligomer present in the samples. Samples were maintained at 4°C until all data had been acquired. A maximum of 10 % oligomer is present in samples of Z AAT. **(h-k)** Diffusion coefficients of M AAT (individual donor), M AAT (8 pooled donors), Z AAT (13 pooled donors) and S AAT (individual donor) respectively measured over the course of acquisition by ^{1}H STE diffusion experiments. Shaded regions for **(h)** M AAT and **(j)** Z AAT **(j)** show the point of addition of 716 ligand. Error bars represent SE of fit from dosyView. Signal intensities measured by ^{1}H 1D NMR (-1 to 1 ppm methyl region shown) for **(l)** M AAT, **(m)** Z AAT, and **(n)** S AAT at the start (blue) and end (red) of acquisition in the ligand free form. ^{1}H 1D NMR spectra (-1 to 1 ppm methyl region) in the presence of the 716 ligand are shown for **(o)** M AAT and **(p)** Z AAT. Blue spectra show signal intensities at the start of ligand addition (following acquisition in the ligand free form) and the red spectra show the signal at the end of acquisition in the presence of the ligand. **(q)** Protease inhibitory activity of AAT samples measured as stoichiometry of inhibition (SI) (see Materials and Methods) pre- and post-NMR acquisition, but prior to 716 addition. Data are represented as mean and standard deviation of 3 technical replicates (individual data points shown as triangles) from individual NMR samples. Source data are provided as a Source Data file.

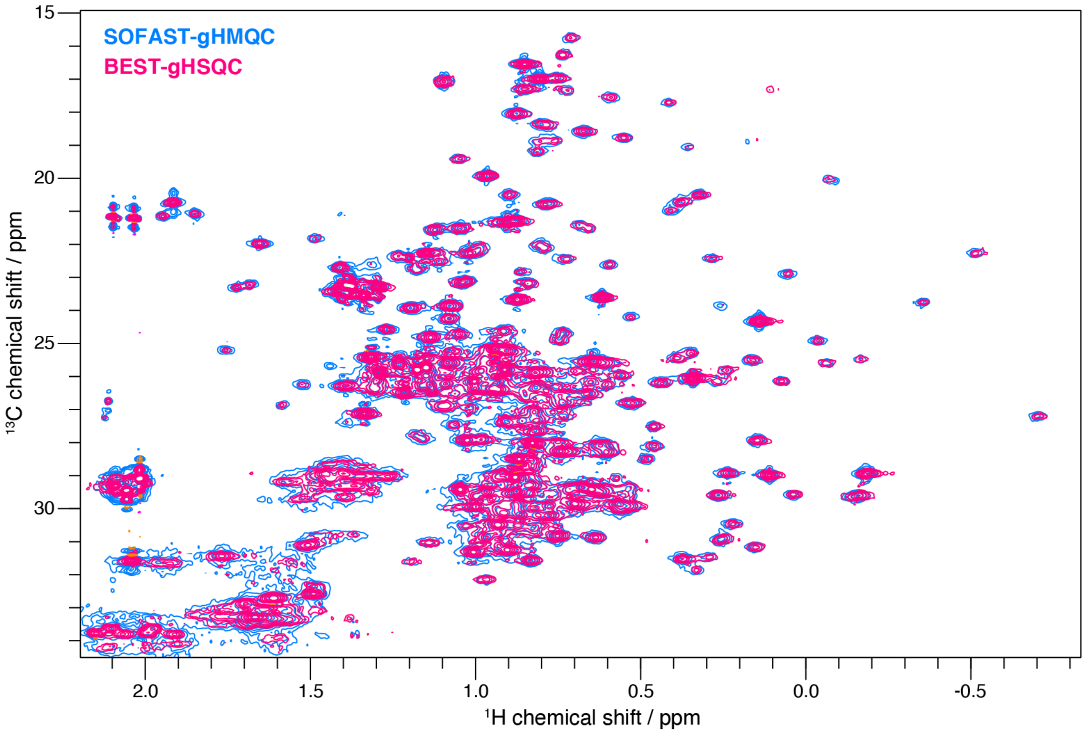

Fig. Supplementary 4: Comparison of ^{1}H,^{13}C HMQC and HSQC experiments for *ex vivo* NMR spectroscopy of AAT. 2D correlation spectra are plotted for a sample of M AAT, acquired using SOFAST-gHMQC and BEST-gHSQC experiments at 298 K, 900 MHz, with identical acquisition times in t_1 and t_2, and using optimized recycle delays. Contour levels have been normalized according to the total acquisition time.

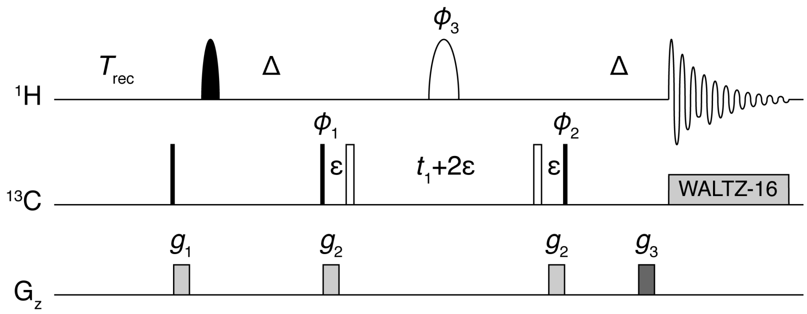

Fig. Supplementary 5: ^{1}H,^{13}C gradient-selected SOFAST-HMQC pulse sequence used for acquisition of *ex vivo* AAT spectra. Filled and open rectangles indicate 90° and 180° ^{13}C pulses. The filled ^{1}H shaped pulse is a 120° PC9 (1.18 ms at 900 MHz) and the open ^{1}H pulse is a 180° r-SNOB (595 µs at 900 MHz), applied at 0.5 ppm in both cases. 3.2 kHz WALTZ-16 decoupling was applied during acquisition. Delays Δ = 1/(2J) = 2 ms and ε = 450 µs. Gradients were applied as smoothed squares: g_1 (1000 µs, 31%), g_2 (250 µs, 40%) and g_3 (250 µs, 20.1%). The phase cycling employed is as follows: φ_1=x, $-x$; φ_2=x, x, $-x$, $-x$; φ_3=x_4, y_4, $(-x)_4$, $(-y)_4$; φ_{rx}=x, $-x$, $-x$, x, $-x$, x, x, $-x$. Echo/anti-echo quadrature detection was achieved by inversion of the sign of g_3 and 180° incrementation of phase φ_1.

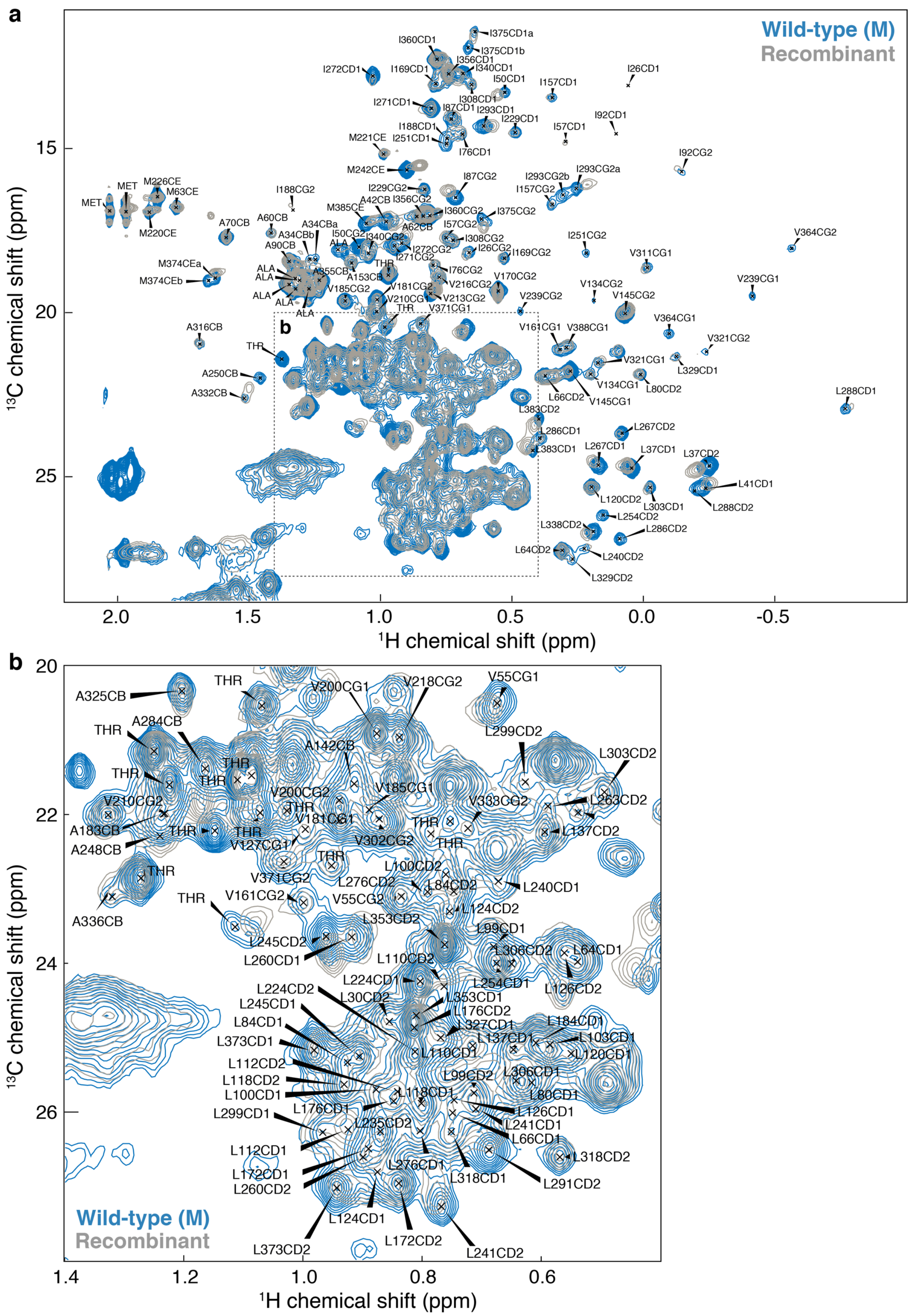

Fig. Supplementary 6: Methyl resonance assignments in *ex vivo* M AAT. (a) ^{1}H,^{13}C SOFAST-gHMQC spectra of wild-type M AAT from a single donor (blue) and U-^{1}H,^{13}C recombinant AAT (grey) (298 K, 900 MHz) with *ex vivo* assignment attributions shown. **(b)** Zoomed region of spectra shown in (a). Residues marked ALA or THR show alanine and threonine residues respectively for which an assignment is unknown.

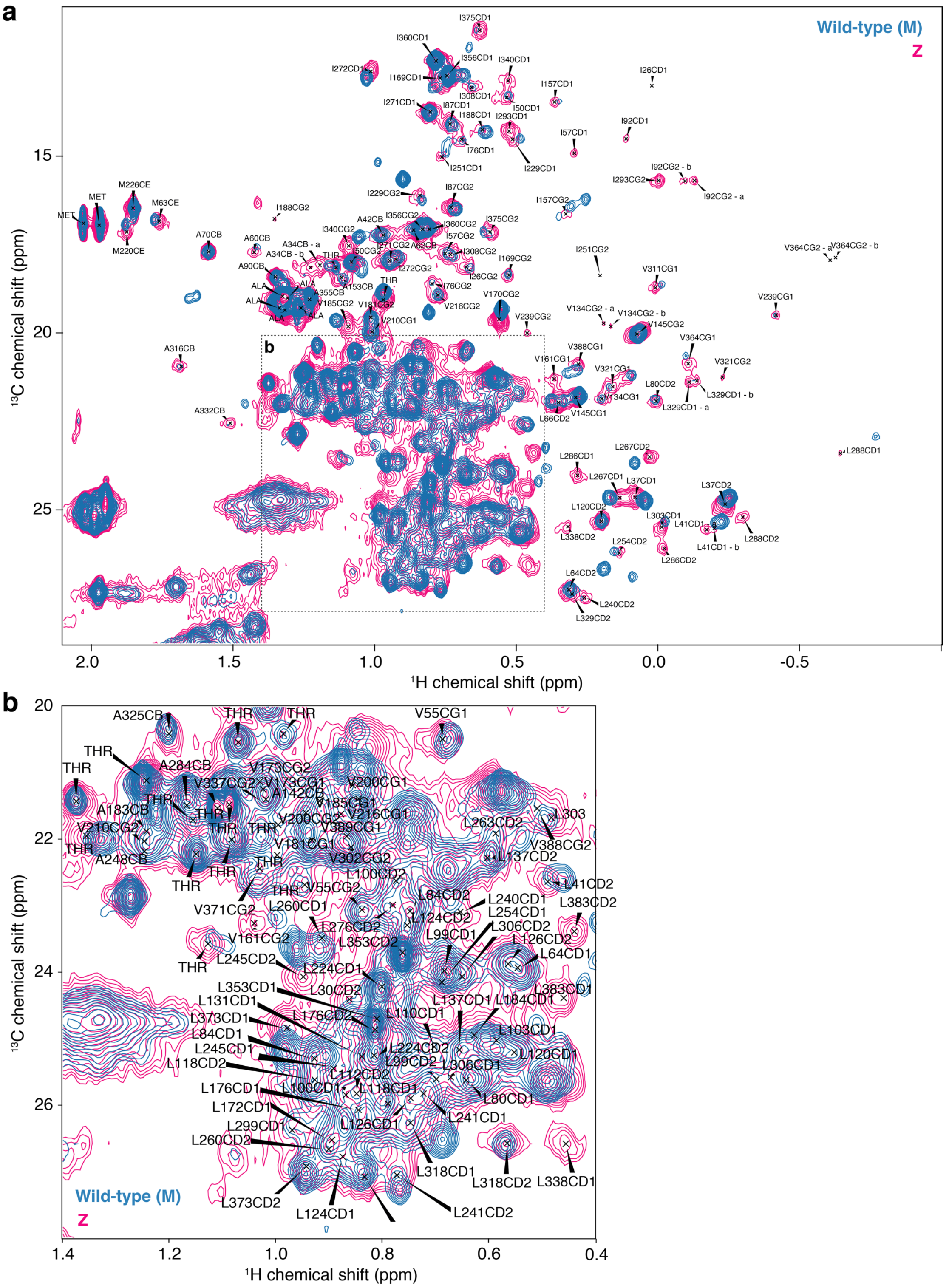

Fig. Supplementary 7: Methyl resonance assignments in *ex vivo* Z AAT. (a) ^{1}H,^{13}C SOFAST-gHMQC spectra of Z AAT from 13 donors (pink) and M AAT (blue) (298 K, 900 MHz) with *ex vivo* assignment attributions for Z AAT shown. **(b)** Zoomed region of spectra shown in (a). Residues marked ALA or THR show alanine and threonine residues respectively for which an assignment is unknown.

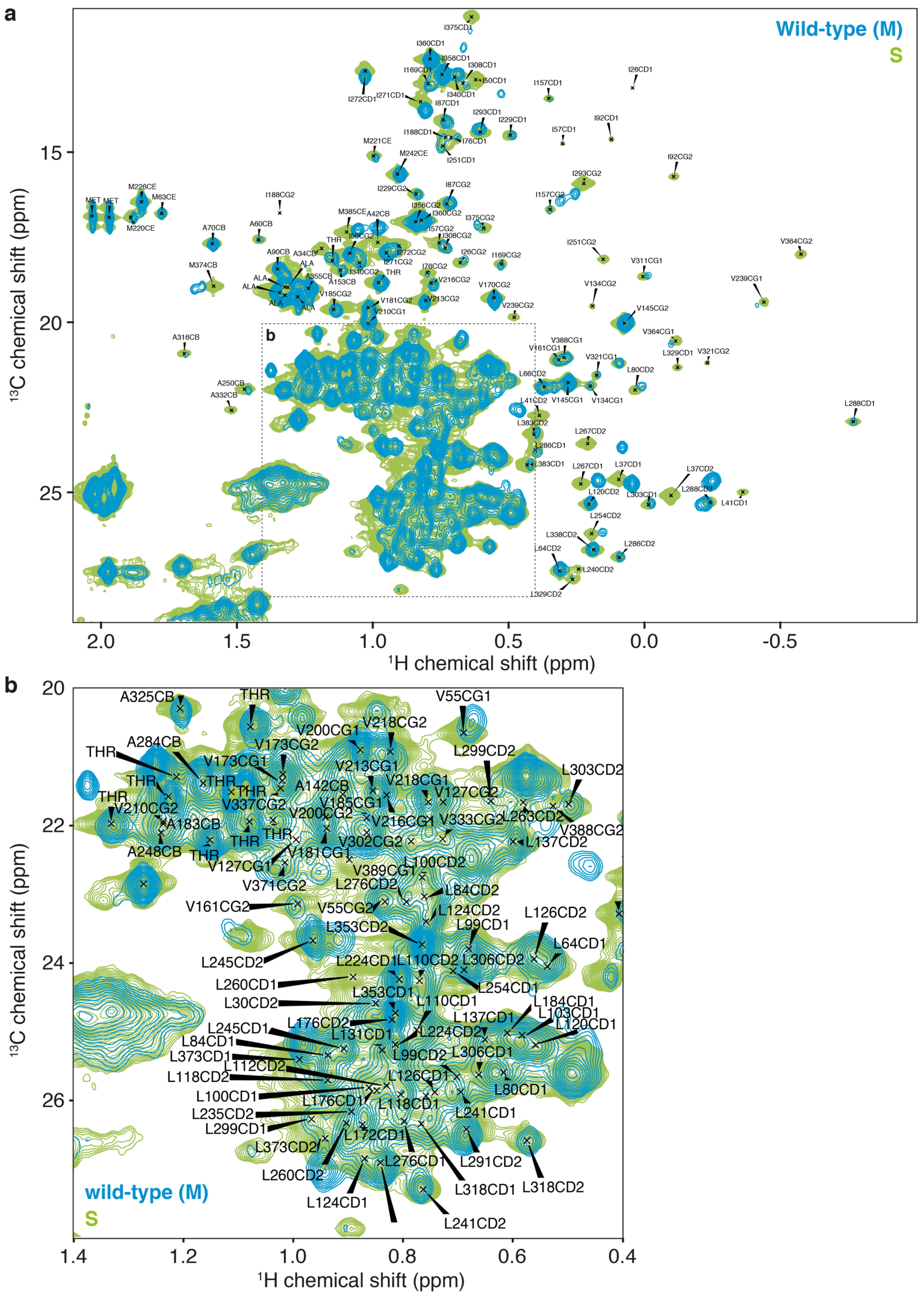

Fig. Supplementary 8: Methyl resonance assignments in *ex vivo* S AAT. (a) ^{1}H,^{13}C SOFAST-gHMQC spectra of S AAT (green) and M AAT (blue) (298 K, 900 MHz) with *ex vivo* assignment attributions for Z AAT shown. **(b)** Zoomed region of spectra shown in (a). Residues marked ALA or THR show alanine and threonine residues respectively for which an assignment is unknown.

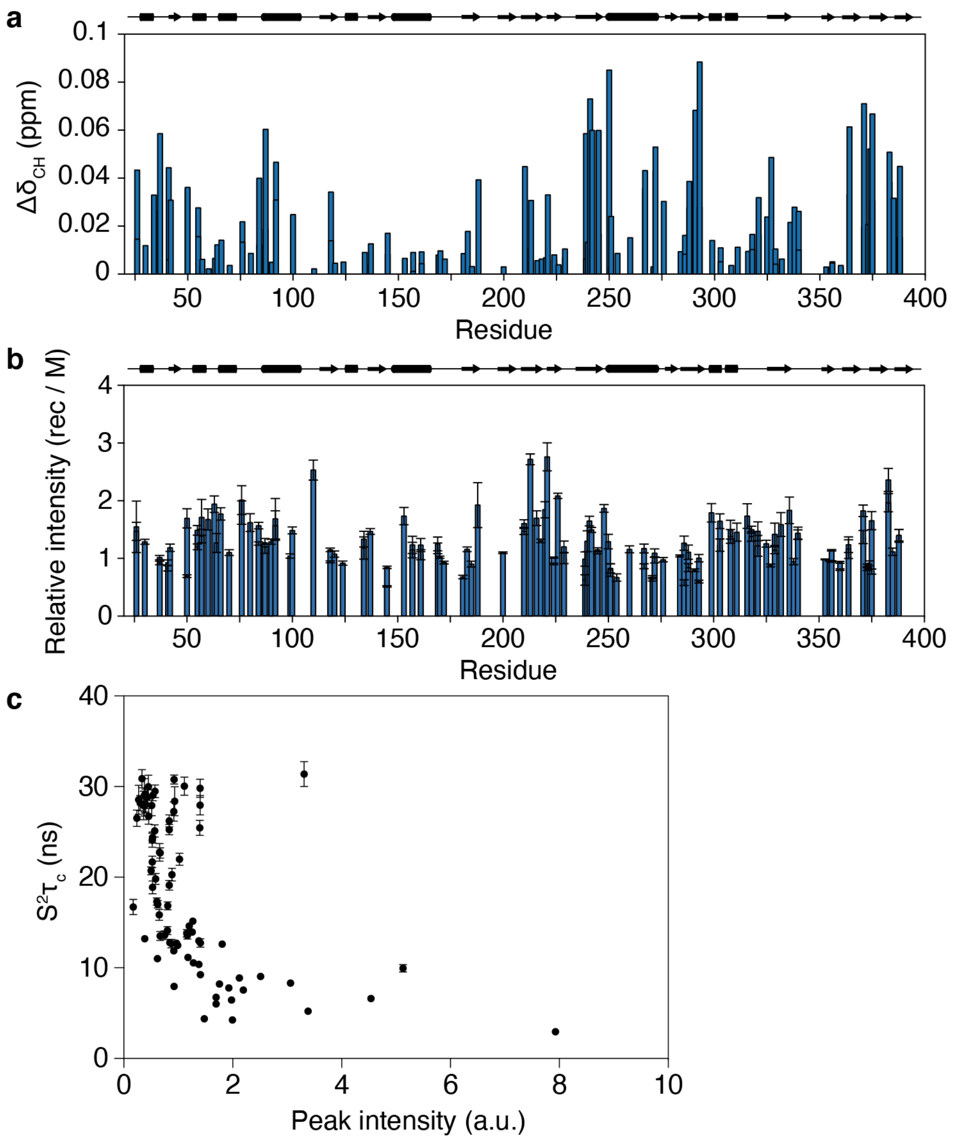

Fig. Supplementary 9: Comparison of *ex vivo* M AAT with labeled recombinant WT AAT.

(a) Chemical shift perturbations of $^1H,^{13}C$ WT AAT determined relative to M AAT, $\Delta\delta_{CH} = \sqrt{\Delta\delta_H^2 + (\Delta\delta_C/4)^2}$. Schematic above represents secondary structure, where cylinders represent alpha helices, arrows represent β-sheets and lines represent unstructured loop regions. **(b)** Relative resonance intensities of $^1H,^{13}C$ WT AAT determined relative to M AAT. Error bars indicate the standard error determined from the spectrum noise level. **(c)** Comparison of $S^2\tau_C$ determined from PLAM labeled recombinant samples with resonance intensities determined from spectra of M AAT. Error bars indicate standard error of fit. Source data are provided as a Source Data file.

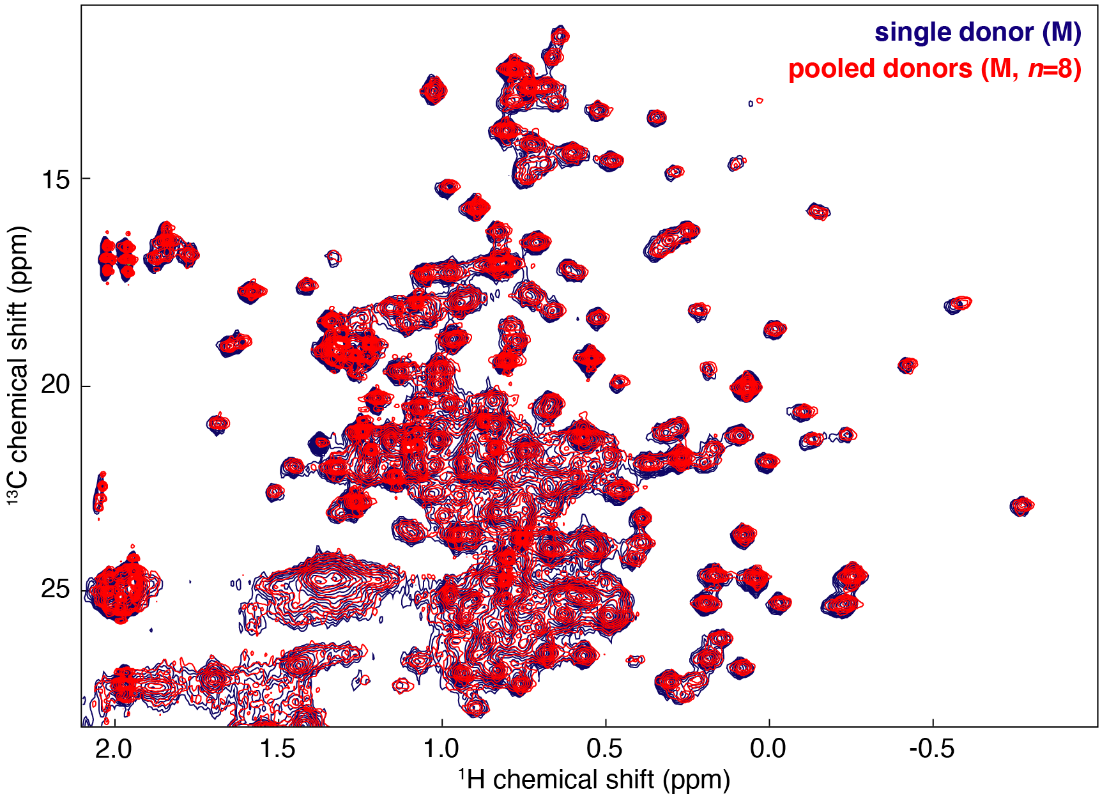

Fig. Supplementary 10. Comparison of $^{1}H,^{13}C$ SOFAST-gHMQC spectra of wild-type M AAT purified from a single donor and from a pool of 8 donors. Contour levels have been normalised according to concentration and acquisition time.

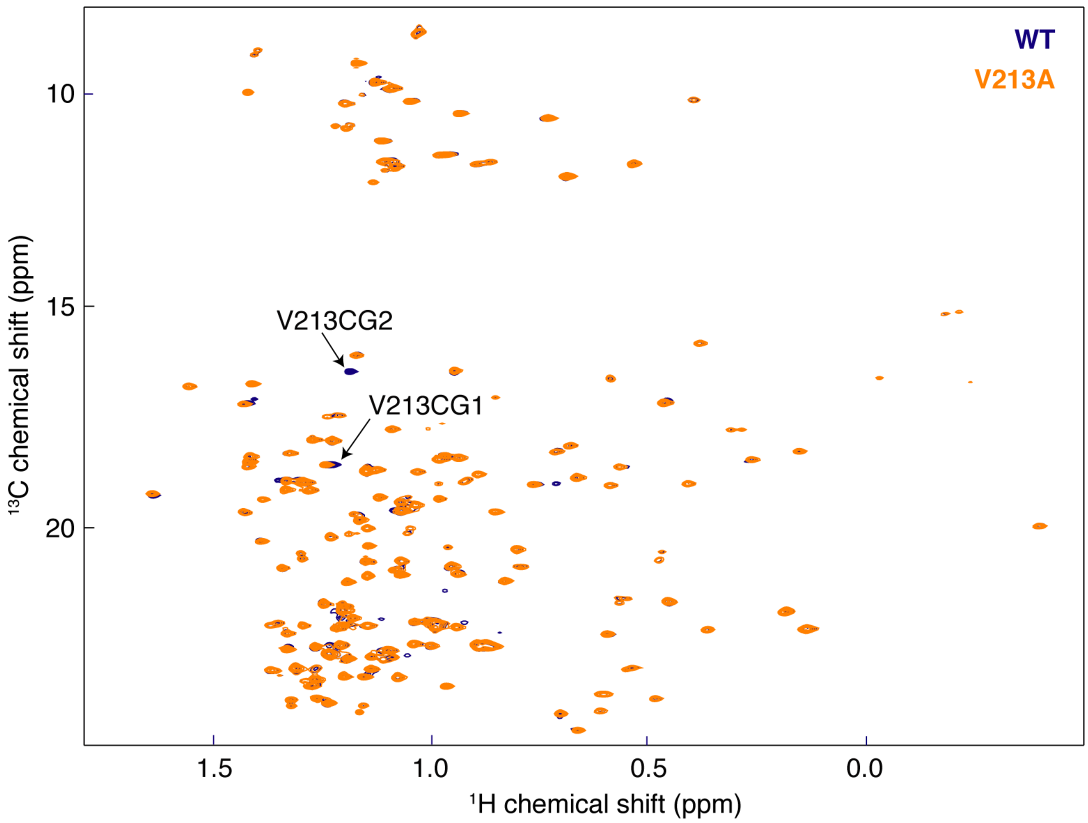

Fig. Supplementary 11. Effect of V231A mutation on AAT spectra. ^{1}H,^{13}C HMQC correlation spectra of recombinant, [^{2}H,^{13}CH$_3$-ILV]-labelled wild-type and V231A AAT are plotted, normalised for concentration and acquisition time. The position of V213 resonances are indicated.

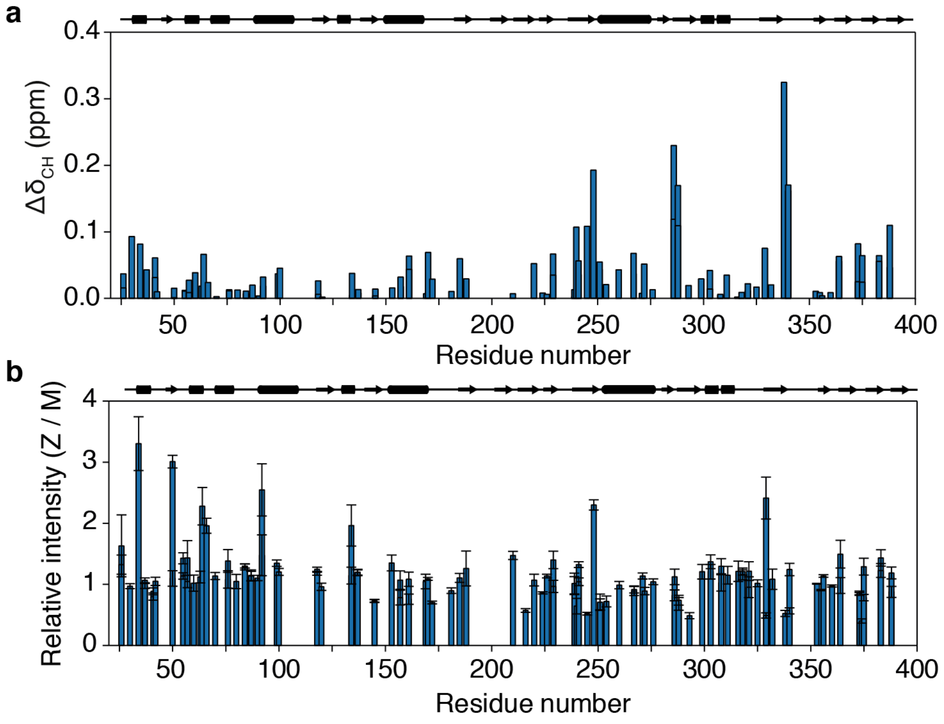

Fig. Supplementary 12. Comparisons of *ex vivo* Z AAT with *ex vivo* M AAT. (a) Chemical shift perturbations of Z AAT determined relative to M AAT, $\Delta\delta_{CH} = \sqrt{\Delta\delta_H^2 + (\Delta\delta_C/4)^2}$. **(b)** Relative resonance intensities of Z AAT determined relative to *ex vivo* M AAT. Error bars indicate the standard error determined from the spectrum noise level. Source data are provided as a Source Data file.

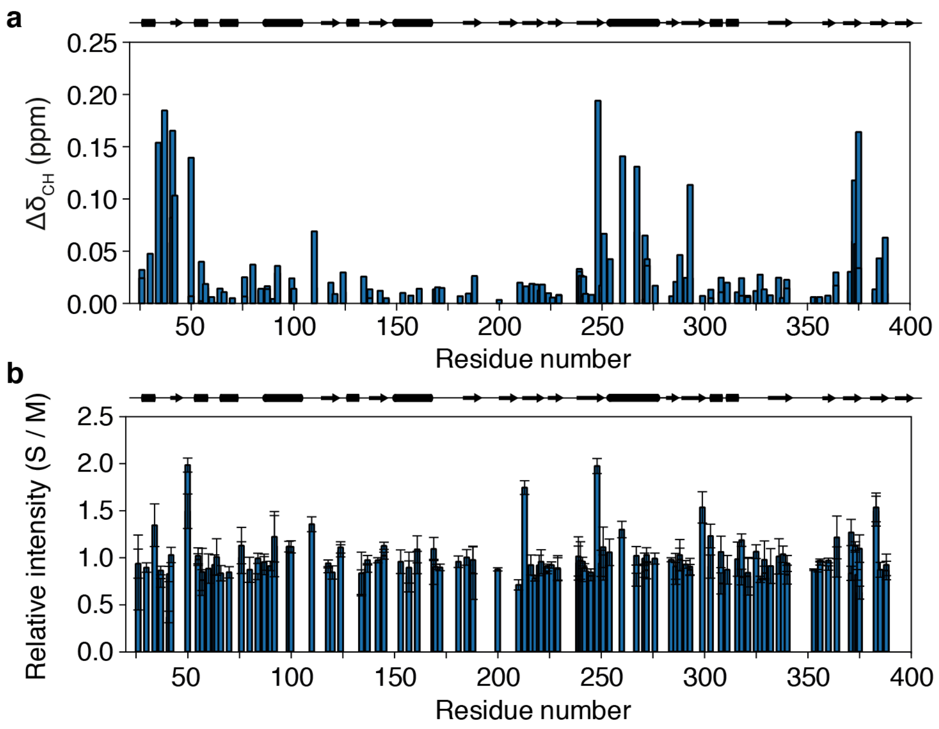

Fig. Supplementary 13: Comparisons of *ex vivo* S AAT with *ex vivo* M AAT. (c) Chemical shift perturbations of S AAT determined relative to M AAT, $\Delta\delta_{CH} = \sqrt{\Delta\delta_H^2 + (\Delta\delta_C/4)^2}$. **(d)** Relative resonance intensities of S AAT determined relative to *ex vivo* M AAT. Error bars indicate the standard error determined from the spectrum noise level. Source data are provided as a Source Data file.

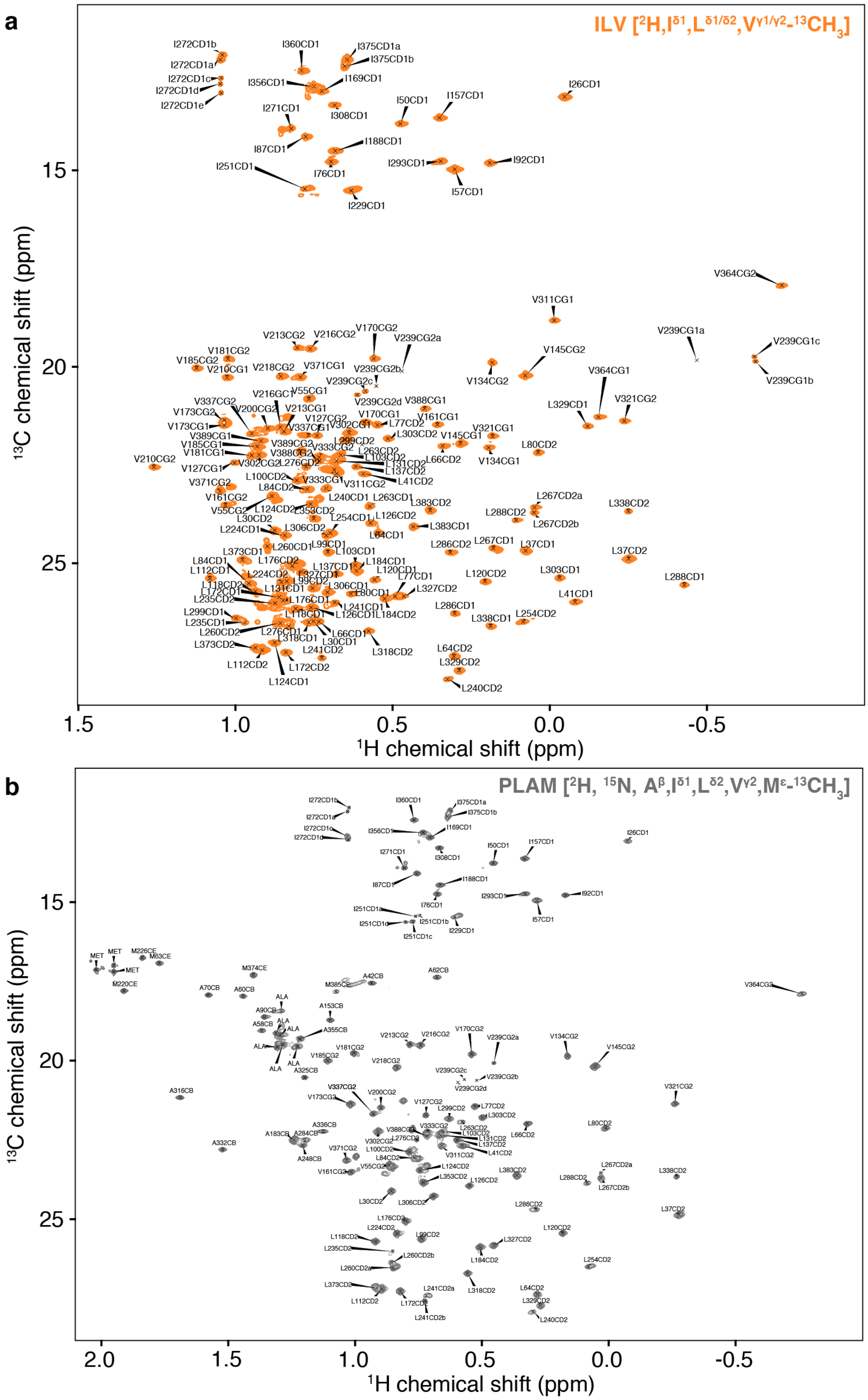

Fig. Supplementary 14: Assignment of WT recombinant AAT bound to the 716 ligand. (a) ^{1}H,^{13}C HMQC spectra of [^{2}H, I$^{\delta1}$L$^{\delta1/\delta2}$,V$^{\gamma1/\gamma2}$-^{13}CH$_3$] (ILV) labeled AAT in the presence of 2 fold molar excess of 716 with assignments shown (298K, 800 MHz). Peaks labelled with residue type mark positions where residue type is known

but assignment is not. Assignments followed by a letter show positions of residue splitting. **(b)** ^{1}H,^{13}C HMQC spectrum of [^{2}H, ^{15}N, $A^{\beta}I^{\delta 1}L^{\delta 2}M^{\varepsilon}V^{\gamma 2}$-$^{13}CH_3$] (PLAM) labeled recombinant AAT in the presence of 2 fold molar excess of 716 (298 K, 800 MHz) with assignments shown. Peaks labelled with residue type mark positions where the residue type is known but an assignment is not. Assignments followed by a letter show positions of residue splitting.

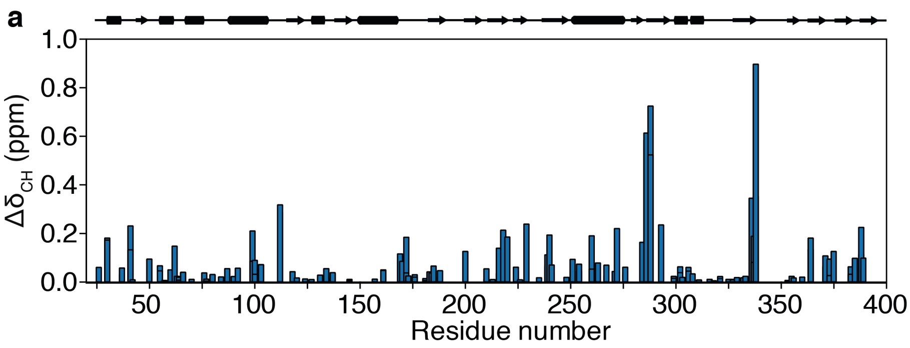

Fig. Supplementary 15: Chemical shift perturbations of recombinant WT AAT upon 716 binding. Combined methyl chemical shift changes,

$\Delta\delta_{CH} = \sqrt{\Delta\delta_H^2 + (\Delta\delta_C/4)^2}$, observed between [^{2}H, ILV-$^{13}CH_3$]-labeled and [^{2}H, ^{15}N, $A^{\beta}I^{\delta 1}L^{\delta 2}M^{\varepsilon}V^{\gamma 2}$-$^{13}CH_3$]-labeled AAT in free and ligand bound states. Source data are provided as a Source Data file.

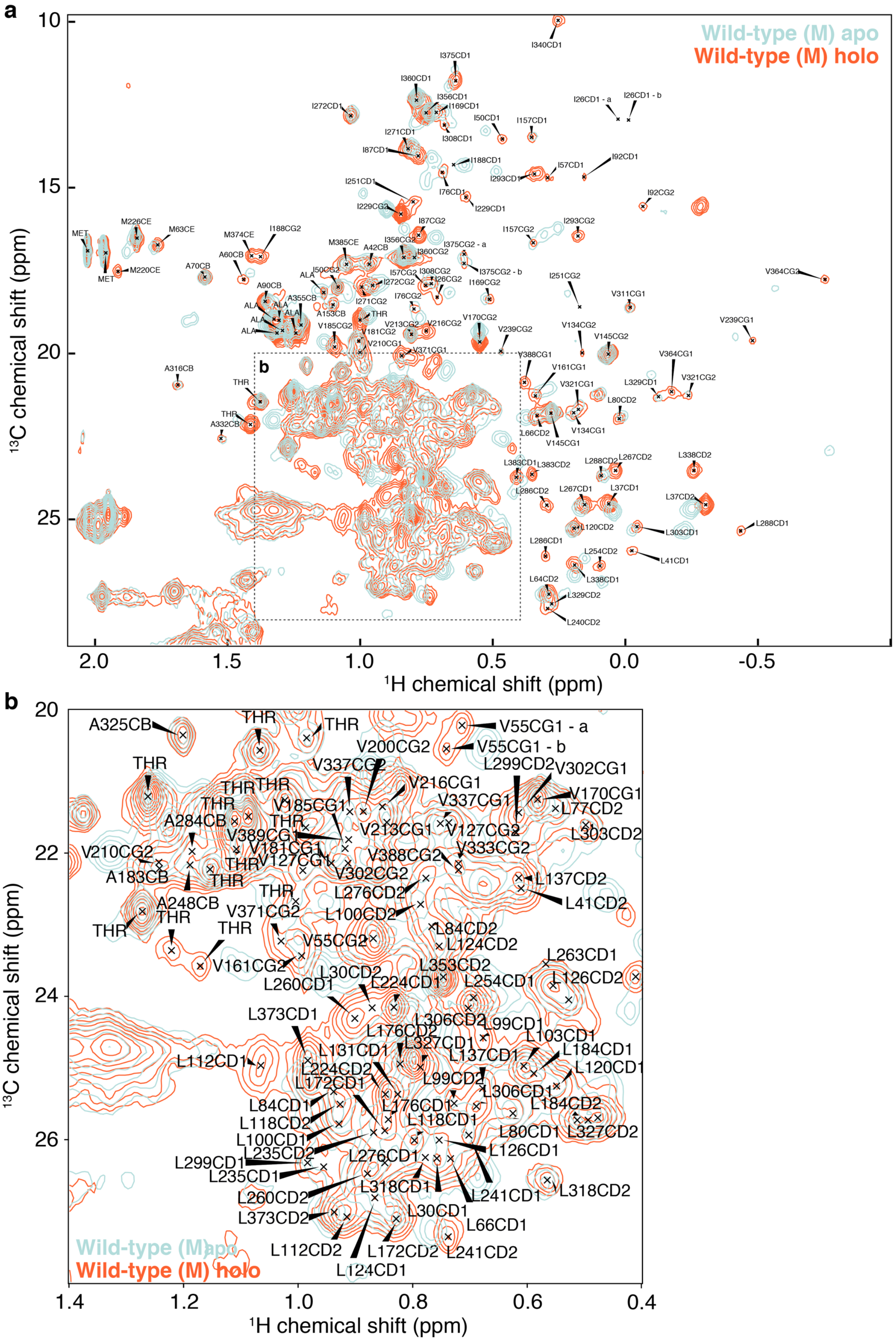

Fig. Supplementary 16: Spectral changes observed in *ex vivo* M AAT upon 716 binding. (a) $^{1}H,^{13}C$ SOFAST-gHMQC spectra of M AAT in absence of 716 ligand (*apo*) (light blue) and M AAT in presence of 2 fold molar excess of 716 ligand (red) (298 K, 900 MHz) with *ex vivo* assignment attributions for M AAT in the presence of 716 shown. **(b)** Zoomed region of spectra shown in (a).

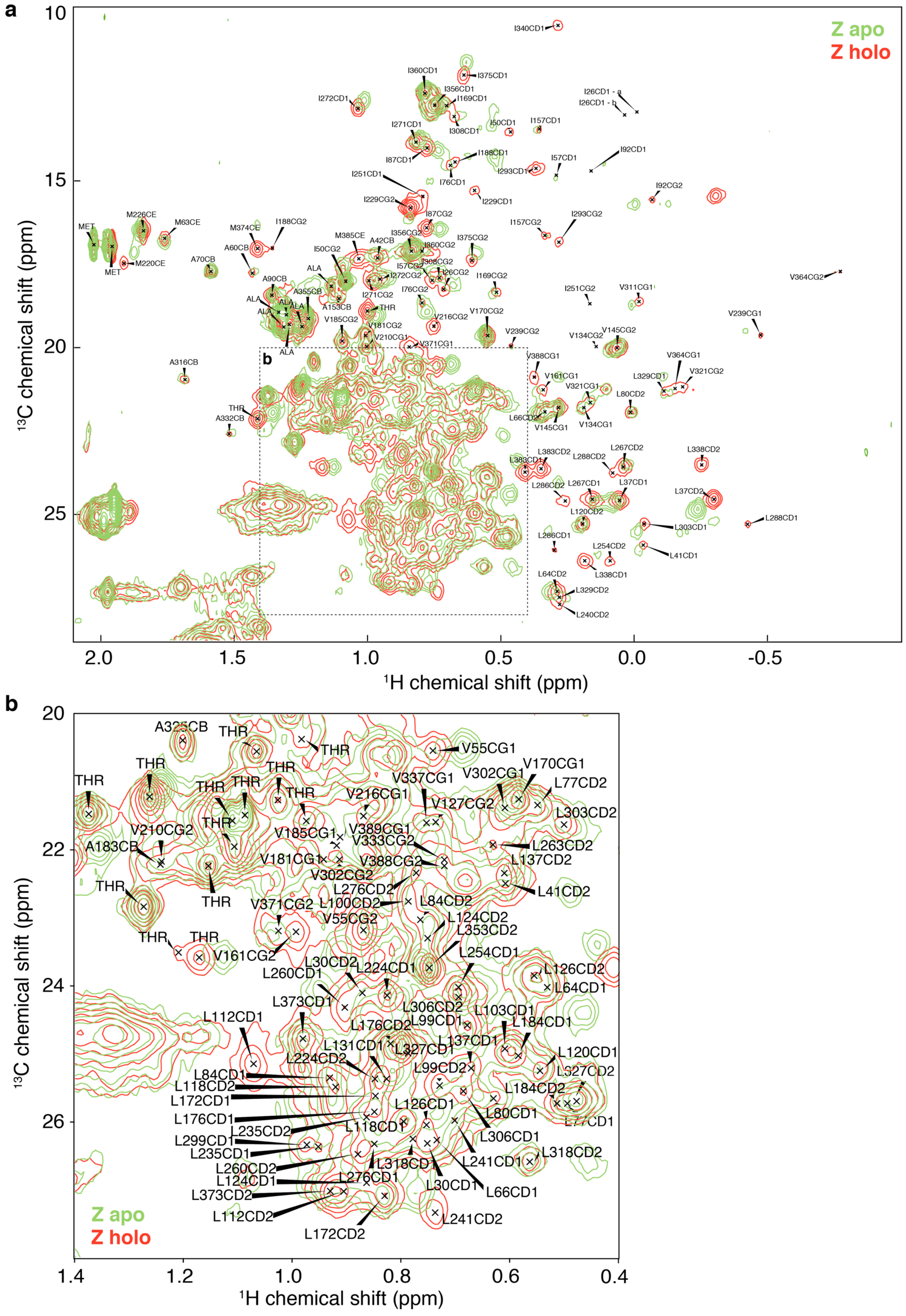

Fig. Supplementary 17: Spectral changes observed in *ex vivo* Z AAT upon 716 binding. (a) ^{1}H,^{13}C SOFAST-gHMQC spectra of Z AAT in absence of 716 ligand (*apo*) (green) and Z AAT in presence of 2 fold molar excess of 716 ligand (red) (298 K, 900 MHz) with *ex vivo* assignment attributions for Z AAT in the presence of 716 shown. **(b)** Zoomed region of spectra shown in (a).

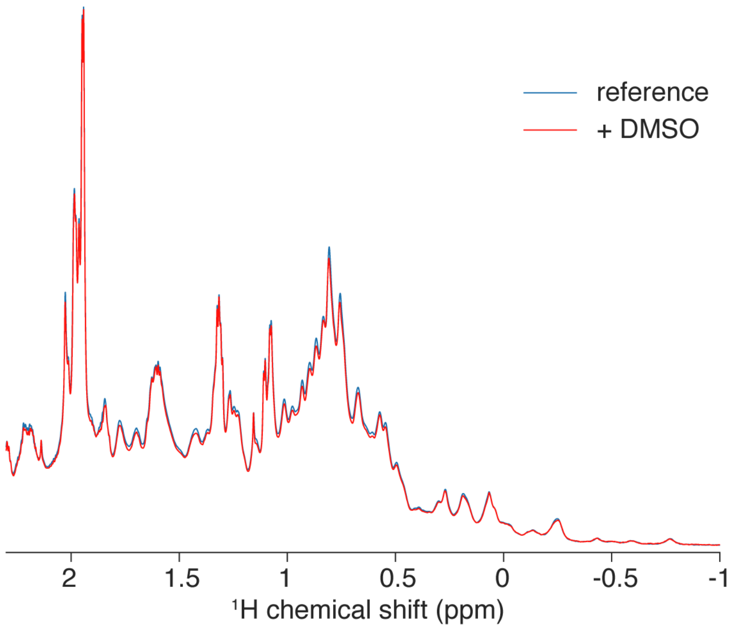

Fig. Supplementary 18: DMSO vehicle control for 716 binding measurements. ^{1}H 1D spectra of M AAT acquired in the presence and absence of 1.7% DMSO.
