# Supplementary material for: High-resolution ex vivo NMR spectroscopy of human Z α1-antitrypsin
Source: Nat Commun. 2020 Dec 11;11:6371. doi: 10.1038/s41467-020-20147-7 (PMC7732992; doi:10.1038/s41467-020-20147-7)
Supplement: Supplementary file 3 — Reporting Summary [file 41467_2020_20147_MOESM3_ESM.pdf]

nature research

Corresponding author(s): Professor David Lomas

Last updated by author(s): Nov 4, 2020

Reporting Summary

Nature Research wishes to improve the reproducibility of the work that we publish. This form provides structure for consistency and transparency in reporting. For further information on Nature Research policies, see our Editorial Policies and the Editorial Policy Checklist.

Statistics

For all statistical analyses, confirm that the following items are present in the figure legend, table legend, main text, or Methods section.

n/a	Confirmed	
☐	☒	The exact sample size (*n*) for each experimental group/condition, given as a discrete number and unit of measurement
☐	☒	A statement on whether measurements were taken from distinct samples or whether the same sample was measured repeatedly
☒	☐	The statistical test(s) used AND whether they are one- or two-sided<br>*Only common tests should be described solely by name; describe more complex techniques in the Methods section.*
☒	☐	A description of all covariates tested
☒	☐	A description of any assumptions or corrections, such as tests of normality and adjustment for multiple comparisons
☐	☒	A full description of the statistical parameters including central tendency (e.g. means) or other basic estimates (e.g. regression coefficient) AND variation (e.g. standard deviation) or associated estimates of uncertainty (e.g. confidence intervals)
☒	☐	For null hypothesis testing, the test statistic (e.g. *F*, *t*, *r*) with confidence intervals, effect sizes, degrees of freedom and *P* value noted<br>*Give P values as exact values whenever suitable.*
☒	☐	For Bayesian analysis, information on the choice of priors and Markov chain Monte Carlo settings
☒	☐	For hierarchical and complex designs, identification of the appropriate level for tests and full reporting of outcomes
☒	☐	Estimates of effect sizes (e.g. Cohen's *d*, Pearson's *r*), indicating how they were calculated

Our web collection on statistics for biologists contains articles on many of the points above.

Software and code

Policy information about availability of computer code

Data collection	Bruker TopSpin 3.6 was used for NMR data acquisition.
Data analysis	Analysis of NMR data used NMRPipe version 9.9, CcpNMR Analysis Version 2.4 Release 2, NMRFAM-SPARKY Version 1.414 and MATLAB R2015b. Analysis of NMR data generated for resonance assignment was non-uniformly sampled. This data was reconstructed using SMILE2.0b following recommended protocols. Data were then visualised using MATLAB 2015b with custom processing scripts (available on request). Preliminary resonance assignments were obtained using the Methyl Assignment by Graph Matching (MAGMA) (no version history) software. GraphPad Prism 6 was used for analysis and representation of protease inhibitory activity data and measurements obtained from NMR data.

For manuscripts utilizing custom algorithms or software that are central to the research but not yet described in published literature, software must be made available to editors and reviewers. We strongly encourage code deposition in a community repository (e.g. GitHub). See the Nature Research guidelines for submitting code & software for further information.

Data

Policy information about availability of data

All manuscripts must include a data availability statement. This statement should provide the following information, where applicable:

- Accession codes, unique identifiers, or web links for publicly available datasets
- A list of figures that have associated raw data
- A description of any restrictions on data availability

The data that support the findings of this study are available from the corresponding author on reasonable request. MATLAB processing scripts can be found at https://github.com/chriswaudby/4d-viewer. Chemical shift assignments, and time-domain NMR data for ex vivo samples have been deposited in the BMRB with the accession codes 50530 to 50536. Protein structures used in this study can be found in the PDB with accession codes 1QLP and 7AEL.

Field-specific reporting

Please select the one below that is the best fit for your research. If you are not sure, read the appropriate sections before making your selection.

☒ Life sciences ☐ Behavioural & social sciences ☐ Ecological, evolutionary & environmental sciences

For a reference copy of the document with all sections, see nature.com/documents/nr-reporting-summary-flat.pdf

Life sciences study design

All studies must disclose on these points even when the disclosure is negative.

Sample size	Biological replicates of ex vivo samples were determined by the availability of patient source material. Due to the large sample requirements of the NMR techniques employed, for rare variants specimens were pooled from multiple donors. The close correspondence between results obtained from single or pooled samples was directly addressed using the wild-type M variant. NMR data acquisition was continued until signal-to-noise levels of at least 3 were obtained for all resonances. Technical replicates for biochemical measurements were performed in triplicate, which was found to provide acceptable standard errors for the purposes of monitoring sample integrity.
Data exclusions	Methyl peaks from overlapping regions of ex-vivo NMR spectra were excluded from analysis.
Replication	Two samples of M AAT, one sample of S AAT and one sample of Z AAT are reported here under optimised and identical acquisition conditions. However, over the entire course of the study, one additional sample of M AAT and one additional sample of Z AAT were acquired, and these also provided extremely similar spectra.
Randomization	Samples were not randomised for this study due to the small number of specialised samples that were prepared.
Blinding	Sample blinding was not relevant given the ability of NMR to identify the sample from the spectrum.

Reporting for specific materials, systems and methods

We require information from authors about some types of materials, experimental systems and methods used in many studies. Here, indicate whether each material, system or method listed is relevant to your study. If you are not sure if a list item applies to your research, read the appropriate section before selecting a response.

Materials & experimental systems

n/a	Involved in the study	
☒	☐	Antibodies
☒	☐	Eukaryotic cell lines
☒	☐	Palaeontology and archaeology
☒	☐	Animals and other organisms
☐	☒	Human research participants
☒	☐	Clinical data
☒	☐	Dual use research of concern

Methods

n/a	Involved in the study	
☒	☐	ChIP-seq
☒	☐	Flow cytometry
☒	☐	MRI-based neuroimaging

Human research participants

Policy information about studies involving human research participants

Population characteristics	See Methods: Preparation of ex vivo AAT; for ex vivo M AAT, plasma from anonymised healthy donors was used. For M AAT pooled samples, plasma from 8 anonymised healthy donors was used. For ex vivo S AAT samples, plasma from one patient determined to be homozygous for the E264V mutation was used. For Z AAT pooled samples, plasma from 13 patients determined to be homozygous for the E324K mutation was pooled and used.
Recruitment	Samples were collected based on patients attending a specialised Alpha-1 Antitrypsin deficiency clinic at Royal Free Hospital, London, with appropriate genotypes.
Ethics oversight	Targeting Dysfunctional Mechanisms in Alpha-1-Antitrypsin Deficiency<br>REC reference: 13/LO/1085<br>NHS Health Research Authority NRES Committee London - Hampstead

Note that full information on the approval of the study protocol must also be provided in the manuscript.
