# Supplementary material for: High-resolution ex vivo NMR spectroscopy of human Z α1-antitrypsin
Source: Nat Commun. 2020 Dec 11;11:6371. doi: 10.1038/s41467-020-20147-7 (PMC7732992; doi:10.1038/s41467-020-20147-7)
Supplement: Supplementary file 2 — Peer Review File [file 41467_2020_20147_MOESM2_ESM.pdf]

Reviewer #1 (Remarks to the Author):

This manuscript reports on an investigation of the effects of mutations on the structure and equilibrium distribution of conformational states of α₁-antitrysin (AAT) by NMR spectroscopy. Taking advantage of the high sensitivity of modern NMR instruments, the investigators are able to obtain spectra for the low abundance 13C signals of AAT methyl groups from samples extracted from the plasma of patients. In order to assign these signals to specific methyl groups in the protein so that the effects on structure and population of conformational states, the authors carry out an extensive series of NMR experiments on isotope-enriched recombinant AAT and its variants. There are two highly significant elements in this report: (1) demonstrating that it is possible to use NMR spectroscopy to study proteins extracted directly from patients; (2) the detection of an intermediate conformational state that is highly populated by a disease associated AAT variant and stabilized by an AAT-directed inhibitor of pathological AAT mis-folding. Both of these elements are of interest to others in the AAT field, and potentially the larger biomedical field.

The experiments were performed to the highest technical standard and represent and particularly effective (albeit highly time and resource demanding) application of solution NMR. [The manuscript would benefit from being a bit more upfront about what conditions are required for this approach to work.] The approach is very well designed to address the specific problem of understanding the structural effects of AAT mutations. Although the detection of 13C (and 15N) NMR signals at natural abundance from biological macromolecules is far from new, the application to proteins extracted from patients is. While one can argue that AAT is a highly favorable case, this does not diminish the fact that there is a need to alert the broader medical/clinical community to the power of this approach. In this sense, one might consider that publication in a journal that directly addresses this community would be more advisable.

The detection of an intermediate conformational state of a protein and increased population in certain variants is also not particularly new. However, the work on AAT and direct characterization of the pathogenic Z variant is. Moreover, the stabilization of this state by the AAT misfolding inhibitor is particularly novel and exciting. As the authors note, these results suggest an interesting new paradigm for treatment of disorders arising from protein mis-folding.

Despite great enthusiasm for the science, there are very substantial issues with the manuscript in its current form. In fact, it is difficult to be certain that all of what is written above is completely accurate due to the lack of clarity and detail in the text. Extensive use of jargon and simple citation of past literature without explanation of the points being made by citing that literature makes the text extremely difficult to follow. This in turn leads one to wonder if the statements made and conclusions drawn are indeed accurate. In short, the manuscript needs extensive revision and clarification, even to fully evaluate the reliability of the results and validity of conclusion. In the following I first list major concerns, then minor issues.

Major concerns

1. Some parts of the Abstract are imprecise and misleading. Limit the hyperbole, especially in the last two sentences. The NMR approach was not developed here; 'develop' should be revised to apply, utilize or demonstrate the applicability of. In the next line and in the last sentence, it is absolutely critical to state it is the NMR of methyl groups being detected. Separate, and provide more detail, for the two major ideas that are presented in the next to last sentence. Connect the intermediate state in Z AAT to misfolding. If a new paradigm is mentioned, then it needs to be stated what it is.

2. Page 5. No interpretation is provided for what rotational diffusion and millisecond dynamics mean in the context of the preceding text. What does this information imply about the protein? The last sentence requires much more information to be clear. Presumably, assignments were first obtained using non-glycosylated recombinant AAT that was enriched in NMR active isotopes, then these were

transferred to the native protein. If not, there is a major concern about the approach.

3. Page 7. The last sentence is extremely difficult to follow. Spin systems are not defined. What are the post-translational modifications expected? The structure of the second half of the sentence is unclear, and it is therefore uncertain if the interpretation is accurate.

4. Page 8. The second half of the bottom paragraph has a series of sentences that are highly opaque. The statements need to be more explicit and even though the results are published, the information that was learned need to be described so that it is possible to understand how these findings relate to the new data reported here. In the absence of this information, it is not possible to fully assess the novelty of results reported here.

5. Page 9, second paragraph. The third sentence is very hard to follow and encompasses too many aspects. Explain in more detail. Presumably, in order to interpret CSPs the resonances observed for GSK 716-bound AAT were assigned first using a sample of isotope-enriched recombinant protein. If not, there is uncertainty about the accuracy of the results. The last sentence is long and difficult to understand. The existence of double peaks for the M sample comes without any previous mention and no indication as to what is their origin. This could conceivably invalidate the conclusions drawn.

6. Page 11. The last two paragraphs are unclear to the point it is difficult to assess the accuracy of the results obtained. Substantially more insights into the procedures need to be provided. What do collinear CSPs mean in this context? State how and what this term means using direct language that relates to the AAT system, not the jargon of protein NMR. The statement that GSK716 does not bind to the native state and stabilizes an intermediate conformation is a huge leap and requires detailed explanation. Without this, it is impossible to determine if this key statement is accurate. Similarly, the extraction of free energies needs to be explained in detail with direct language and description of specific data obtained.

7. Is the experiment shown as Figure 2 Extended Materials somehow unique? If so, this needs to be explained in the text. If not, the figure is not needed and the relevant acquisition parameters should be included in the Methods section.

Additional points

1. State the origin of the odd nomenclature (M, Z, S) for AAT and its variants. The native protein M seems to be considered a variant in this terminology. Since the system is opaque, some explanation would be useful for the broader audience.

2. Page 5. In the current sentence structure, it is not clear why the interleaved 1D and diffusion measurements were performed.

3. Figure 2, panel C needs to be replotted using colors that are sufficiently different from each other to detect differences in the data.

4. Page 9, second paragraph, third line. Preferentially is imprecise and misleading, use “with high affinity”.

5. Recommend switching the order of the two paragraphs in the Discussion. The last sentence of the second paragraph should be deleted as it is pure speculation.

Reviewer #2 (Remarks to the Author):

This manuscript describes an ex vivo NMR approach applied to wild-type (M) and pathogenic variants (Z and S) of the protein alpha-1-antitrypsin (AT). The approach is interesting and novel in the context of understanding AT misfolding and polymerization. The authors suggest long-range conformational/dynamical changes in the region of helix F caused by the Z mutation. In addition, they demonstrate that a compound that binds to a region near the Z mutation stabilizes a non-native state that is in equilibrium with the native state in all forms of AT. They conclude that this state is an intermediate on the pathway to misfolding.

Although the approach is interesting and of potential value in understanding the structure and dynamics of AT, the conclusions do not appear supported by the data, and the manuscript is written in a confused and often overstated manner. The latter is evident throughout, from the title suggesting a high resolution structure, to the discussion concluding identifying the 'transient species responsible for a serpin-mediated conformational disorder'. This and other conclusions appear to be unfounded, and are not argued for but rather stated.

Although the technique is of interest in context of AT misfolding, the conclusions are not justified by the data presented. I would recommend the authors consider re-writing this study to provide a simple description of the data, and discuss more dispassionately the possible interpretations.

Reviewer #3 (Remarks to the Author):

This manuscript presents an important and excellently conducted study to examine the structure and biophysical properties of near-native AAT protein, obtained and purified directly from consenting patients, to resolve critical questions regarding the role and mechanism in COPD. The study presents a careful stepwise characterization of the protein(s) to enable the use of recombinant protein to validate and support data when the ex vivo proteins are not available in sufficient quantities or sensitivity limits some experiments. The protocols are both clever and expertly designed, leading to confidant conclusions that had escaped the field previously.

The careful analysis of residue specific information allows this study to resolve several controversies and disparate interpretations of the interactions between AAT variants and the small molecule inhibitor, 716. The ability to monitor subtle effects through chemical shift perturbations and dynamics illustrates the power of NMR in such near native conditions. The data are convincing that there are not significant structural modifications between the ground state and the intermediate state that recognizes and binds the 716 inhibitor. This is a completely reasonable finding, and it emphasizes the roles of dynamics and minor state populations in the regulation of important biological processes.

The authors are to be commended for an elegant and rigorous approach to collecting and analyzing data for native/patient protein samples at natural abundance and the skillful interleaved use of recombinant isotopically labeled samples. The combination provides powerful and unique insight that will have clear implications for understanding disease.

The biological impact is high, illustrating that ex vivo samples can be analyzed with amino acid resolution in very complex structures. This is both equal to advanced cryoEM and should be noted that it will work in concert with cryoEM as such studies advance. The advantage of the NMR tools is that this enables the sampling of minor states that actually are the recognition points. Such analyses, when used in combination with tools such as cryoEM, will forge new frontiers in understanding biology.

Minor corrections:

1. Figure 1 legend: The abbreviations of M, Z, S variants are used prior to description. Please consider how to establish these prior to using them in the text/legends.

2. The introduction of the term ex vivo occurs on page 4, yet it is not clear to what level of concentration the protein is increased for the NMR studies. => data in Extended Figs 3.

3. It is not always clear if the sample is recombinant or ex vivo. Recommend being more specific in the text to emphasize the points. For example, at the top of page 7, the comparison is made between "Z and S variants.. with with that of the wild-type (M)". It is clear that Z and S are ex vivo but it is not clear if "wild-type M" is recombinant. It must be assumed from the legend of Figure 2, that "wild-type M" is also ex vivo.

4. Legend to Figure 3. The use of dark red and magenta to highlight different characteristics is a poor choice, particularly when the figure will be small in print. A different contrast would be beneficial.

5. It would be appropriate to cite a reference for the diffusion experiments used in the Materials and Methods, page 23.

6. The separation of the references from the normal location to after the Materials and Methods as 'Additional References' is unusual. If these are to go with the Extended or Supporting information, then perhaps this is acceptable. It is up to the editors.

7. Figure 4D. It would be advantageous to indicate helix F in this image. It is assumed that the color scheme for the ribbon diagram is consistent with Figure 1. Perhaps this consistency of coloring can be stated at the time of discussing Figure 1 and the structure. It could also be annotated in Extended figure 9, where the secondary structure does not have the consistent coloring as in Figure 1 and 4.

Reviewer #1 (Remarks to the Author):

This manuscript reports on an investigation of the effects of mutations on the structure and equilibrium distribution of conformational states of α₁-antitrypsin (AAT) by NMR spectroscopy. Taking advantage of the high sensitivity of modern NMR instruments, the investigators are able to obtain spectra for the low abundance 13C signals of AAT methyl groups from samples extracted from the plasma of patients. In order to assign these signals to specific methyl groups in the protein so that the effects on structure and population of conformational states, the authors carry out an extensive series of NMR experiments on isotope-enriched recombinant AAT and its variants. There are two highly significant elements in this report: (1) demonstrating that it is possible to use NMR spectroscopy to study proteins extracted directly from patients; (2) the detection of an intermediate conformational state that is highly populated by a disease associated AAT variant and stabilized by an AAT-directed inhibitor of pathological AAT mis-folding. Both of these elements are of interest to others in the AAT field, and potentially the larger biomedical field.

We are grateful to the reviewer for the accurate summary of our work, and the recognition of the significance of our key findings.

The experiments were performed to the highest technical standard and represent and particularly effective (albeit highly time and resource demanding) application of solution NMR. [The manuscript would benefit from being a bit more upfront about what conditions are required for this approach to work.] The approach is very well designed to address the specific problem of understanding the structural effects of AAT mutations. Although the detection of 13C (and 15N) NMR signals at natural abundance from biological macromolecules is far from new, the application to proteins extracted from patients is. While one can argue that AAT is a highly favorable case, this does not diminish the fact that there is a need to alert the broader medical/clinical community to the power of this approach. In this sense, one might consider that publication in a journal that directly addresses this community would be more advisable.

As suggested, we have now included a detailed discussion of the optimisation of experimental conditions, acquisition time and sample requirements within the Results section of the revised manuscript (p. 6–8). In particular, we now clearly describe the

necessary sample concentration (400 uM), temperature (298 K) and NMR acquisition time (ca. 80 hr) that was required for these measurements. We have also now included references to both historic and modern applications of natural abundance NMR to biomolecules, at the opening of the Results section (p. 5, final paragraph), and again in the Discussion (p. 21, first paragraph).

While the reviewer suggests that a journal addressing the medical and clinical community directly might be appropriate, we respectively disagree: we feel that the broad readership of *Nature Communications* makes it a highly suitable journal for this work, which we hope will be of interest to NMR spectroscopists, structural biologists, medicinal chemists in addition to medics and clinicians.

The detection of an intermediate conformational state of a protein and increased population in certain variants is also not particularly new. However, the work on AAT and direct characterization of the pathogenic Z variant is. Moreover, the stabilization of this state by the AAT misfolding inhibitor is particularly novel and exciting. As the authors note, these results suggest an interesting new paradigm for treatment of disorders arising from protein mis-folding.

Despite great enthusiasm for the science, there are very substantial issues with the manuscript in its current form. In fact, it is difficult to be certain that all of what is written above is completely accurate due to the lack of clarity and detail in the text. Extensive use of jargon and simple citation of past literature without explanation of the points being made by citing that literature makes the text extremely difficult to follow. This in turn leads one to wonder if the statements made and conclusions drawn are indeed accurate. In short, the manuscript needs extensive revision and clarification, even to fully evaluate the reliability of the results and validity of conclusion. In the following I first list major concerns, then minor issues.

We regret that our initial submission has been unclear and difficult to follow. In spite of concerns to the contrary, we do feel that the reviewer has indeed presented a fair and accurate summary of our work, for which we are grateful. In our revised submission, we have substantially rewritten the Introduction and (particularly) the Results section to provide more background and detail on literature cited, improved explanations of complex NMR terminology to minimise jargon, and generally to provide more detail and clarity on the experiments we have carried out. In doing so,

we have split up and re-structured several figures to bring forward key data that was previously in supporting data (e.g. resonance assignments in the revised Figure 3), and to provide an illustration of the key concept of collinear chemical shift perturbations (Fig. 7b).

Major concerns

1. Some parts of the Abstract are imprecise and misleading. Limit the hyperbole, especially in the last two sentences. The NMR approach was not developed here; 'develop' should be revised to apply, utilize or demonstrate the applicability of. In the next line and in the last sentence, it is absolutely critical to state it is the NMR of methyl groups being detected. Separate, and provide more detail, for the two major ideas that are presented in the next to last sentence. Connect the intermediate state in Z AAT to misfolding. If a new paradigm is mentioned, then it needs to be stated what it is.

We have substantially revised the Abstract to address the reviewer's concerns. We have eliminated the final sentence, while the penultimate sentence has been revised to remove 'For the first time…'. We have altered 'develop' to 'apply' as suggested (l. 9). We have clarified that it is methyl groups that are being observed ('We observed widespread chemical shift perturbations *for methyl groups* in spectra of Z AAT, l. 11–12). We have introduced the connection between intermediate formation and misfolding ('misfolding is generally accepted to occur via a transiently populated intermediate', l. 6–7), and re-written the final sentences to separate (i) the identification of the intermediate state in Z AAT, and (ii) the new paradigm that stabilisation of a disease-associated state provides for the treatment of protein misfolding disorders. The core concept of this new paradigm is also now stated explicitly in the abstract as suggested ('wherein the stabilisation of disease-associated intermediate states provides a basis for selectivity while inhibiting further transitions along misfolding pathways').

2. Page 5. No interpretation is provided for what rotational diffusion and millisecond dynamics mean in the context of the preceding text. What does this information imply about the protein? The last sentence requires much more information to be clear. Presumably, assignments were first obtained using non-glycosylated recombinant AAT that was enriched in NMR active isotopes, then these were transferred to the native protein. If not, there is a major concern about the approach.

In light of the reviewer's comments, we have completely re-written the discussion of resonance intensities, to introduce the key concepts such as rotational diffusion, chemical exchange and millisecond-timescale dynamics (p. 12, last paragraph):

> Lastly, we examined the variation in resonance intensities between methyl groups in M AAT. Such site-to-site variations reflect differences in transverse relaxation rates, which in turn are determined primarily by the local proton density, the rate of rotational diffusion (represented by the rotational correlation time, τ_c), and the local mobility at a particular site (represented by an S^2 order parameter), although chemical exchange (i.e. exchange between multiple conformations having distinct chemical shifts) on an approximately micro–millisecond timescale may also contribute to increased relaxation rates and hence reduced resonance intensities. Therefore, the analysis of resonance intensities provides a valuable tool for probing conformational dynamics and the potential existence of intermediate states.

We have also comprehensively re-written our discussion of resonance assignments. As the reviewer notes, having correct assignments is absolutely critical to our approach and to the correct interpretation of our results. It is an area that we have taken very seriously and indeed that has required several years of work. While these details were previously provided in the methods section and as supplementary figures, in our revised manuscript we have introduced a new sub-section within the Results section, *'Assignment of AAT methyl resonances'* (p. 8), in which we describe in detail the assignment of recombinant AAT (which is now presented clearly in a main figure, Fig. 3), and then the transfer of these assignments to *ex vivo* spectra of AAT variants.

3. Page 7. The last sentence is extremely difficult to follow. Spin systems are not defined. What are the post-translational modifications expected? The structure of the second half of the sentence is unclear, and it is therefore uncertain if the interpretation is accurate.

We have completely re-written this section (p. 13–15) to clarify our experimental approach and expand on the discussion of our results, avoiding the use of NMR jargon such as 'spin systems' in favour of 'methyl groups'. The post-translational modifications that are mentioned refers to N-linked glycosylation, which occurs at

three sites within AAT. We have now included an initial discussion of glycosylation and glycan heterogeneity within a substantially revised and more detailed comparison of *ex vivo* M AAT spectra with the recombinant wild-type (p. 11 last paragraph–p. 12 second paragraph), which in turn leads to a clearer discussion of differences in glycan heterogeneity observed between the M and Z AAT variants (p. 14, first paragraph).

4. Page 8. The second half of the bottom paragraph has a series of sentences that are highly opaque. The statements need to be more explicit and even though the results are published, the information that was learned need to be described so that it is possible to understand how these findings relate to the new data reported here. In the absence of this information, it is not possible to fully assess the novelty of results reported here.

We have completely re-written this section, in order to improve the clarity and provide more detail and background to the reader (p. 13–15). In particular, we have clarified the literature references, which referred to the conformational changes undergone by AAT during both function and polymerisation: 'This region is a central component of the serpin inhibitory apparatus, as large conformational changes must occur in both the F-helix and β-sheet A to enable insertion of the RCL within the sheet, and it has also been implicated by multiple studies in the polymerisation mechanism' (p. 14, final paragraph).

5. Page 9, second paragraph. The third sentence is very hard to follow and encompasses too many aspects. Explain in more detail. Presumably, in order to interpret CSPs the resonances observed for GSK 716-bound AAT were assigned first using a sample of isotope-enriched recombinant protein. If not, there is uncertainty about the accuracy of the results. The last sentence is long and difficult to understand. The existence of double peaks for the M sample comes without any previous mention and no indication as to what is their origin. This could conceivably invalidate the conclusions drawn.

We have completely re-written and expanded this section to provide greater detail and clarity (p. 15–17). As the reviewer has correctly inferred, references of 716-bound AAT were assigned initially using recombinant samples of AAT. While this was described originally in the methods section, we now describe this explicitly within the Results section of the revised manuscript (p. 17, first paragraph). We have also

split Fig. 4 in the original manuscript into two new figures, in which the assignment and analysis of chemical shift perturbations in recombinant AAT are presented explicitly (Fig. 6), and then in which *ex vivo* spectra are subsequently analysed (Fig. 7).

The reference to double peaks refers to the observation of a small number of multiple resonances in spectra of (unbound) M AAT, adjacent to the N247 glycosylation site and which we attribute to perturbations in the local chemical environment that arise from covalently distinct glycoforms. This is a subject that we have now addressed in much greater detail in the revised manuscript: in the original comparison of M AAT with recombinant AAT (p. 12, second paragraph), in the comparison of M and Z AAT (p. 14, first paragraph), and in the analysis of 716 binding (p. 17, second paragraph).

6. Page 11. The last two paragraphs are unclear to the point it is difficult to assess the accuracy of the results obtained. Substantially more insights into the procedures need to be provided. What do collinear CSPs mean in this context? State how and what this term means using direct language that relates to the AAT system, not the jargon of protein NMR. The statement that GSK716 does not bind to the native state and stabilizes an intermediate conformation is a huge leap and requires detailed explanation. Without this, it is impossible to determine if this key statement is accurate. Similarly, the extraction of free energies needs to be explained in detail with direct language and description of specific data obtained.

We have substantially revised and expanded this section to provide additional detail and clarity (p. 19–20). We have provided an explicit definition of collinearity: ‘CSPs observed between M and Z AAT were collinear with those observed upon binding of 716, with resonances of Z AAT observed at an approximately constant fraction of the distance between the unbound (M) and bound resonances’ (p. 19, l. 7–9). We have created a new figure panel, Fig. 7b, that illustrates the key concepts of the analysis, and an additional equilibrium scheme, Fig. 7d, that explicitly depicts the conformational selection model to which data are fitted. Referring to these new figure panels, we have provided a new, detailed explanation of the mechanistic origin of collinear chemical shift perturbations that makes clear the justification for our conclusions (p. 19, paragraphs 2 and 3):

> Collinear, correlated CSPs are a powerful and unambiguous indicator of conformational exchange and arise where a rapid equilibrium exists between

> two conformations with distinct chemical shifts, such that the observed chemical shift is the population-weighted average of the individual states. Modulation of this equilibrium, either by mutation or by ligand binding, thus results in correlated perturbations across many resonances, which in 2D spectra gives rise to collinear shift perturbations (Fig. 7b)[56]. […]
>
> In the case of AAT, as 716 binding is observed to shift the equilibrium towards the intermediate state (Fig. 7a) we conclude that 716 does not bind to the native state but must instead interact through a conformational selection mechanism (Fig. 7d), in which an intermediate conformation that is spontaneously sampled by AAT is stabilised by ligand binding. In this model, the ligand has a constant affinity for the intermediate state across all variants, and so the apparent macroscopic affinity is determined only by the equilibrium population of the intermediate in the absence of the ligand. As we observe the population of this intermediate to be significantly increased by the Z mutation, this successfully accounts for the observed increase in the macroscopic affinity of 716 for the Z variant (Fig. 6b).

We have also expanded significantly on the fitting of the observed results to the conformational selection model (p. 20, first paragraph):

> To quantify the intermediate populations in AAT variants, we fitted our observations of relative CSPs, together with measurements of 716 binding affinities (Fig. 6b), to a conformational selection scheme (Fig. 7d) in order to determine both the affinity of 716 for the pure intermediate state, and the relative chemical shift of the pure native state (see Methods). Given the high affinity of the ligand for AAT, together with the fact that binding resulted in very similar chemical shifts for both M and Z AAT, we have assumed that the bound state shifts are a close approximation to those of the (unbound) intermediate. The populations of the intermediate state at equilibrium could then be determined from the observed CSPs, which also allowed the relative free energies of native (N), intermediate (I) and 716-bound (B) states in M, Z and S AAT to be calculated (Figs. 7e–f).

Mathematical details of this analysis continue to be provided in the Methods section, to which we now explicitly refer the reader in the above text.

7. Is the experiment shown as Figure 2 Extended Materials somehow unique? If so, this needs to be explained in the text. If not, the figure is not needed and the relevant acquisition parameters should be included in the Methods section.

To the best of our knowledge, the gradient-selected SOFAST-HMQC experiment has not been reported previously, and provided essential improvements in the quality of natural abundance spectra we obtained through the elimination of t_1 noise. We have added an explanation of this pulse sequence to the revised Results section (p. 8, first paragraph).

Additional points

1. State the origin of the odd nomenclature (M, Z, S) for AAT and its variants. The native protein M seems to be considered a variant in this terminology. Since the system is opaque, some explanation would be useful for the broader audience.

We have provided an explanation of this nomenclature in our revised Introduction (p. 3, first paragraph):

> The names of common AAT alleles follow an historic nomenclature, in which they are assigned letters based on their migration during isoelectric focussing electrophoresis with respect to the wild-type ‘M’ (medium) allele[17]. In this work, this single-letter nomenclature will refer exclusively to *ex vivo* human material, while recombinant samples will be explicitly labelled as such.

2. Page 5. In the current sentence structure, it is not clear why the interleaved 1D and diffusion measurements were performed.

We have provided a more detailed explanation of the quality control measurements in the revised Results section (p. 6, final paragraph):

> Following protocols developed for the analysis of unstable ribosome–nascent chain complexes[42], ^{1}H 1D and translational diffusion measurements[43] were acquired every 5 hr during the NMR acquisition period to provide a real-time assessment of sample stability (Fig. 2a).

3. Figure 2, panel C needs to be replotted using colors that are sufficiently different from each other to detect differences in the data.

As suggested, we have altered the colours in this figure panel (now Fig. 4c) to red and blue.

4. Page 9, second paragraph, third line. Preferentially is imprecise and misleading, use “with high affinity”.

We have re-phrased this in line with the reviewer’s suggestion (p. 16, l. 1–2):

> This molecule binds close to the site of the Z mutation, E342K, with a significantly higher affinity for Z AAT than for M or S AAT (Fig. 4b–c)

5. Recommend switching the order of the two paragraphs in the Discussion. The last sentence of the second paragraph should be deleted as it is pure speculation.

We agree, and have revised the Discussion accordingly (p. 21–22).

Reviewer #2 (Remarks to the Author):

This manuscript describes an ex vivo NMR approach applied to wild-type (M) and pathogenic variants (Z and S) of the protein alpha-1-antitrypsin (AT). The approach is interesting and novel in the context of understanding AT misfolding and polymerization. The authors suggest long-range conformational/dynamical changes in the region of helix F caused by the Z mutation. In addition, they demonstrate that a compound that binds to a region near the Z mutation stabilizes a non-native state that is in equilibrium with the native state in all forms of AT. They conclude that this state is an intermediate on the pathway to misfolding.

Although the approach is interesting and of potential value in understanding the structure and dynamics of AT, the conclusions do not appear supported by the data, and the manuscript is written in a confused and often overstated manner. The latter is evident throughout, from the title suggesting a high resolution structure, to the discussion concluding identifying the 'transient species responsible for a serpin-

mediated conformational disorder'. This and other conclusions appear to be unfounded, and are not argued for but rather stated.

Although the technique is of interest in context of AT misfolding, the conclusions are not justified by the data presented. I would recommend the authors consider re-writing this study to provide a simple description of the data, and discuss more dispassionately the possible interpretations.

We regret that our original submission was unclear and confusing to the reviewer, although we respectfully disagree with the assertion that our conclusions are not justified by the data we have presented. We have re-written and greatly expanded the Results section of our revised manuscript in order to provide much more detail and clarity.

As noted in our response to reviewer 1's comments, we have substantially revised the Abstract, Introduction and Discussion sections to reduce the 'hype' and present our results in a more dispassionate manner. In particular, in the Discussion we now refer to 'the transient species *associated with* a serpin-mediated conformational disorder' (p. 21, second paragraph).

We believe that our original title, 'High-resolution *ex vivo* NMR spectroscopy of human Z α_1-antitrypsin', remains an accurate description of our work: we make no reference to the determination of a structure, but rather 'high-resolution' is intended to refer to the remarkable quality of 2D NMR spectra that we have obtained from large, unlabelled glycoproteins, that in turn has enabled a series of residue-specific analyses of structural perturbations and dynamics within these molecules. However, if this continues to be a point of concern then we would be willing to consider alternatives.

Reviewer #3 (Remarks to the Author)

This manuscript presents an important and excellently conducted study to examine the structure and biophysical properties of near-native AAT protein, obtained and purified directly from consenting patients, to resolve critical questions regarding the role and mechanism in COPD. The study presents a careful stepwise characterization of the protein(s) to enable the use of recombinant protein to validate

and support data when the ex vivo proteins are not available in sufficient quantities or sensitivity limits some experiments. The protocols are both clever and expertly designed, leading to confidant conclusions that had escaped the field previously.

The careful analysis of residue specific information allows this study to resolve several controversies and disparate interpretations of the interactions between AAT variants and the small molecule inhibitor, 716. The ability to monitor subtle effects through chemical shift perturbations and dynamics illustrates the power of NMR in such near native conditions. The data are convincing that there are not significant structural modifications between the ground state and the intermediate state that recognizes and binds the 716 inhibitor. This is a completely reasonable finding, and it emphasizes the roles of dynamics and minor state populations in the regulation of important biological processes.

The authors are to be commended for an elegant and rigorous approach to collecting and analyzing data for native/patient protein samples at natural abundance and the skillful interleaved use of recombinant isotopically labeled samples. The combination provides powerful and unique insight that will have clear implications for understanding disease.

The biological impact is high, illustrating that ex vivo samples can be analyzed with amino acid resolution in very complex structures. This is both equal to advanced cryoEM and should be noted that it will work in concert with cryoEM as such studies advance. The advantage of the NMR tools is that this enables the sampling of minor states that actually are the recognition points. Such analyses, when used in combination with tools such as cryoEM, will forge new frontiers in understanding biology.

We are grateful for the reviewer’s generous summary of our manuscript. Bearing in mind the additional comments received by the editor directly, we have nevertheless substantially revised the Results section in order to clarify and explain more carefully unfamiliar NMR experiments and jargon, and we hope that our revised manuscript will be more accessible to a broad readership.

Minor corrections:

1. Figure 1 legend: The abbreviations of M, Z, S variants are used prior to description. Please consider how to establish these prior to using them in the text/legends.

We have now split Fig. 1 into two separate figures, which provide a general introduction to AAT (Fig. 1), and then present the *ex vivo* NMR spectra of M, Z and S variants (Fig. 2). In this way, the variant nomenclature will be introduced prior to its appearance in figures and figure legends. We have also added a more detailed explanation of the variant nomenclature in the revised Introduction (p. 4, second paragraph):

> The names of common AAT alleles follow an historic nomenclature, in which they are assigned letters based on their migration during isoelectric focussing electrophoresis with respect to the wild-type 'M' (medium) allele[17]. In this work, this single-letter nomenclature will refer exclusively to *ex vivo* human material, while recombinant samples will be explicitly labelled as such.

2. The introduction of the term ex vivo occurs on page 4, yet it is not clear to what level of concentration the protein is increased for the NMR studies. => data in Extended Figs 3.

We now state clearly the concentrations that are used for *ex vivo* NMR measurements in the Results section, and clarify their relationship to concentrations *in vivo* (p. 6, first paragraph):

> [W]e selected a sample concentration of 400 μM (approximately 10 to 25-fold higher than that of wild-type levels of circulating AAT in human plasma[18]) … No changes were observed between 1D ^{1}H spectra acquired at AAT concentrations of 1 and 400 μM, indicating that the elevated concentrations used in this study have not induced any detectable changes such as oligomerisation (Fig. S2).

3. It is not always clear if the sample is recombinant or ex vivo. Recommend being more specific in the text to emphasize the points. For example, at the top of page 7, the comparison is made between "Z and S variants.. with that of the wild-type (M)". It is clear that Z and S are ex vivo but it is not clear if "wild-type M" is recombinant. It must be assumed from the legend of Figure 2 that "wild-type M" is also ex vivo.

As noted in our response to comment 1 above, we have revised our Introduction (p. 4, second paragraph) to state that we consistently refer to *ex vivo* samples as M, Z and S, while recombinant samples are explicitly labelled as such.

4. Legend to Figure 3. The use of dark red and magenta to highlight different characteristics is a poor choice, particularly when the figure will be small in print. A different contrast would be beneficial.

We have revised this figure (Fig. 5 in the revised manuscript) to use black in place of dark red. We have also increased the size of this key figure to provide additional clarity.

5. It would be appropriate to cite a reference for the diffusion experiments used in the Materials and Methods, page 23.

We have now added an appropriate reference.

6. The separation of the references from the normal location to after the Materials and Methods as 'Additional References' is unusual. If these are to go with the Extended or Supporting information, then perhaps this is acceptable. It is up to the editors.

We believe that we have correctly followed the format requested by *Nature Communications*. However, we are happy to follow the Editor's guidance on this.

7. Figure 4D. It would be advantageous to indicate helix F in this image. It is assumed that the color scheme for the ribbon diagram is consistent with Figure 1. Perhaps this consistency of coloring can be stated at the time of discussing Figure 1 and the structure. It could also be annotated in Extended figure 9, where the secondary structure does not have the consistent coloring as in Figure 1 and 4.

We have indeed used consistent colouring between Figure 1 and Figure 4 (i.e. Figure 6 in the revised manuscript), and we now refer to this in the legend of Fig. 6. The structure in Extended figure 9 has been removed in our revised manuscript, as it is a duplication of Fig. 6e, which is now shown in a larger, clearer format.

Reviewer #1 (Remarks to the Author):

The authors have done an outstanding job of responding to the reviews; the revised manuscript is now clear and on point.

A few minor suggestions are included in the following.

The most significant discovery from this extensive NMR analysis is that GSK716 binds and stabilizes an AAT folding intermediate. The authors are encouraged to include a more substantial discussion of the implications of this finding. An additional paragraph or 3-4 sentences would be sufficient.

Line 104: ... performed TODAY using ...

Line 107-108: ... overlapping SIGNALS (or RESONANCES) using ...

Line 158: cite a reference for natural abundance HMQC having similar resolution and greater sensitivity than HSQC for natural abundance samples.

Line 441: the 2nd standard deviation has two significant figures- be sure to check if this is appropriate.

Reviewer #3 (Remarks to the Author):

The revision by Jagger, Waudby, et al. represents a significant improvement in the writing and communication of what was already an impressive manuscript. The authors have addressed all of the concerns raised by this reviewer and have admirably responded to comments from the other reviewers.

The revision is a strong publication that provides an important demonstration of the power of solution state NMR applied to patient samples. This study provides an important connection between traditional research samples and actual patient samples, thus addressing the direct impact on understanding human biology.

Minor Comment:
1. Page 19, lines 433-444. The description of collinear, correlated CSPs is significantly improved in the revision. Nevertheless, the additional meaning of ‘collinear’ to the description of a fast-exchange, two state system remains slightly opaque. The meaning is discernable from the figure legend of Figure 7C. There may be some consideration given to explaining collinear in the text.

R. Andrew Byrd

Reviewer #1 (Remarks to the Author):

The authors have done an outstanding job of responding to the reviews; the revised manuscript is now clear and on point.

Thank you – we are grateful to the reviewer for the comments prompting these revisions.

A few minor suggestions are included in the following.

The most significant discovery from this extensive NMR analysis is that GSK716 binds and stabilizes an AAT folding intermediate. The authors are encouraged to include a more substantial discussion of the implications of this finding. An additional paragraph or 3-4 sentences would be sufficient.

We welcome the opportunity to discuss the implications of our finding further, and have revised the discussion as suggested (p. 21–22).

Line 104: ... performed TODAY using ...

We have altered the text here (p. 5, 2nd paragraph) as suggested.

Line 107-108: ... overlapping SIGNALS (or RESONANCES) using ...

We have altered the text here (p. 5, 2nd paragraph) as suggested.

Line 158: cite a reference for natural abundance HMQC having similar resolution and greater sensitivity than HSQC for natural abundance samples.

Our original text was unclear on this point: we have in fact directly compared both experiments in order to select empirically the more sensitive method (Fig. S4). We have revised this section to include a more detailed discussion of the balance between an optimal methyl TROSY effect and the impact of interactions with external protons that underlie the choice of HSQC or HMQC experiments, including an additional reference [43] where these effects have originally been discussed. We hope that our revised text now has greater clarity on this point.

Line 441: the 2nd standard deviation has two significant figures- be sure to check if this is appropriate.

We believe in this instance that our original text, 0.004 ± 0.017, is preferable to the alternative, 0.00 ± 0.02 (p. 19, 2nd paragraph). Note that in response to comments from reviewer 3 this section has been revised, and these results are now stated as percentages, 27 ± 2 % and 0.4 ± 1.7 %.

Reviewer #3 (Remarks to the Author):

The revision by Jagger, Waudby, et al. represents a significant improvement in the writing and communication of what was already an impressive manuscript. The authors have addressed all of the concerns raised by this reviewer and have admirably responded to comments from the other reviewers.

We are again grateful to the reviewers for their helpful and constructive comments.

The revision is a strong publication that provides an important demonstration of the power of solution state NMR applied to patient samples. This study provides an important connection between traditional research samples and actual patient samples, thus addressing the direct impact on understanding human biology.

Minor Comment:

1. Page 19, lines 433-444. The description of collinear, correlated CSPs is significantly improved in the revision. Nevertheless, the additional meaning of ‘collinear’ to the description of a fast-exchange, two state system remains slightly opaque. The meaning is discernable from the figure legend of Figure 7C. There may be some consideration given to explaining collinear in the text.

We have revised this section to add further clarification, and to emphasise that is it the correlated nature of chemical shift perturbations across many methyl groups that is the most significant observation: although highly distinctive, collinear shift perturbations are simply a result of these correlated perturbations occurring for both ^{1}H and ^{13}C chemical shifts within a single methyl group. We have therefore renamed the section from ‘Collinear chemical shift perturbations…’ to ‘Correlated chemical shift perturbations…’, and discuss in the first paragraph both the correlation of ^{1}H and ^{13}C CSPs (i.e. collinearity), and the more general correlation of CSPs across many methyl groups. We also now indicate explicitly in the text rather than the figure legend that we are analysing exchange between native and intermediate states, including a reference to fast chemical exchange (which was previously postponed to a later paragraph). Finally, we have revised the discussion of Fig. 7c to clarify the choice of reference points, and to provide an improved description of the linear regression analysis. We hope that the revised text provides much greater clarity to this key section.
